# Cleavage and inactivation of poly(ADP-ribose) polymerase in peripheral blood mononuclear cells of patients with acute respiratory distress syndrome

**DOI:** 10.1186/s12931-026-03623-4

**Published:** 2026-03-16

**Authors:** Sidnéia Sousa Santos, Kelly Ascenção, Karim Zuhra, Vanessa Martins, Giuseppe Gianini Figueiredo Leite, Larissa de Oliveira Cavalcanti Peres Rodrigues, Jackeline Y. Hayashi, Milena Karina Colo Brunialti, Laszlo Pecze, Lucas Liaudet, Jelena Vukovic, Govind Sridharan, Reinaldo Salomão, Csaba Szabo

**Affiliations:** 1 Section of Pharmacology, Department of Oncology, Microbiology and Immunology, Faculty of Science and Medicine, University of Fribourg, Fribourg, 1700 Switzerland; 2 Department of Medicine, Division of Infectious Diseases, Escola Paulista de Medicina, Federal University of São Paulo (EPM/UNIFESP), São Paulo, 04023 Brazil; 3 Service of Adult Intensive Care Medicine, University Hospital Medical Center, Lausanne University, Lausanne, 1015 Switzerland; 4 Section of Medicine, Faculty of Science and Medicine, University of Fribourg, Fribourg, 1700 Switzerland; 5 Department of Intensive Care Medicine, Fribourg Hospital, Fribourg, 1700 Switzerland

**Keywords:** Lung, Bioenergetics, Mitochondria, Cytokines, Oxidative stress, Leukocyte

## Abstract

Olaparib, the first clinically approved poly (ADP-ribose) polymerase (PARP) inhibitor, may be repurposed for non-oncological conditions such as acute respiratory distress syndrome (ARDS), where PARP-1 inhibition has shown benefits in preclinical models. We investigated the expression and functional status of PARP-1 and the effects of olaparib in peripheral blood mononuclear cells (PBMCs) from ARDS patients and healthy controls. PBMCs from healthy volunteers (*N* = 8) and ARDS patients (*N* = 8) were isolated via Ficoll gradient. PARP-1, cleaved PARP (cPARP), and PAR polymers were assessed by Western blotting. Cytokine production was measured in plasma and in PBMC supernatants after 1 h preincubation with olaparib (10 µM) or vehicle, followed by LPS (100 ng/ml) stimulation for 4 h. Cellular bioenergetics were analyzed using Seahorse XFe24 after H_2_O_2_ (100 µM, 2 h) with or without olaparib pretreatment. Control PBMCs showed a lymphocyte-predominant population with mostly full-length PARP-1. In ARDS Day 1 samples, PARylated proteins increased and PARP became downregulated. By Day 8, PARylation decreased and full-length PARP-1, as well as cleaved PARP-1 were detectable. Olaparib treatment of the cells did not alter the LPS-induced cytokine responses. Exposure of healthy PBMCs to oxidative stress suppressed cellular bioenergetics, and this effect was attenuated by olaparib. However, in ARDS PBMCs, which were already bioenergetically suppressed, oxidative stress had no further effect, and olaparib was without protective effect. Thus, in PBMCs isolated from ARDS, olaparib’s cytoprotective effect is no longer detectable, likely due to PARP-1 inactivation and degradation.

**Supplementary Information:**

The online version contains supplementary material available at 10.1186/s12931-026-03623-4.

## Introduction

Acute respiratory distress syndrome (ARDS) is a common cause of respiratory failure in severely ill patients and is defined by the acute onset of non-cardiogenic pulmonary edema, hypoxemia, and the need for mechanical ventilation [6, 17, 42]. The pathophysiology of ARDS includes the activation of inflammation, coagulation and various injury response pathways –– both within the lung and systemically. The activation of the innate immune response triggers the recruitment of neutrophils and monocytes, resulting in the release of various pro-inflammatory mediators, which can trigger local, as well as systemic responses, including the generation of various free radicals, oxidants and other reactive species [24, 42].

Increases in reactive oxygen species generation can culminate in DNA breakage and consequent overactivation of the constitutive mammalian nuclear and mitochondrial enzyme PARP-1. Activated PARP-1, catalyzes a reaction in which the ADP-ribose portion of nicotinamide adenine dinucleotide (NAD^+^) is transferred to a receptor amino acid, building poly (ADP-ribose) (PAR) polymers in a process known as PARylation. PARP activation can lead to mitochondrial dysfunction, cellular ATP depletion, increased mitochondrial reactive oxygen species (ROS) generation, the release of apoptosis-inducing factor (AIF), release of cytochrome c, can induce various forms of cell death, including necrosis and parthanatos [9]. PARP-1 can also stimulate the production of pro-inflammatory mediators and activate various feedforward cycles of cell injury [9, 18, 39, 41]. Several lines of preclinical data – including the results of cell-based models, rodent models and large animal models, utilizing either genetic or pharmacological inhibition of PARP – demonstrate the importance of PARP activation in the pathogenesis of ARDS [27, 41].

Olaparib is the first clinically approved poly (ADP-ribose) polymerase (PARP) inhibitor used in the therapy of ovarian and breast cancer [9]. In addition to its use in oncology, a significant body of preclinical data indicate that it may also be useful in the experimental therapy of ARDS, as it exerts cytoprotective and anti-inflammatory effects (reviewed in [41]). Indeed, the concept of repurposing clinically approved PARP inhibitors for various non-oncological diseases – including sepsis and septic shock, various forms of reperfusion injury, inflammation, fibrosis and neurodegeneration – has emerged in the last decade [4, 21, 30, 39, 47]. In particular, preclinical studies conducted with olaparib, demonstrate that this PARP inhibitor exerts cytoprotective effects both in vitro, in various cell types exposed to oxidative stress [1–3, 22, 27, 31, 35, 38] and in vivo, in various rodent models of ARDS [19, 26, 33, 37]. Among these studies, we have recently demonstrated the protective effect of olaparib at low concentrations against oxidative stress in human peripheral blood leukocytes, with recovery of NAD^+^ mitochondrial membrane potential and ATP levels [38]. The same study also demonstrated that olaparib does not impair the generation of microbicidal reactive species and does not inhibit the cells' phagocytic activity after bacterial challenge [38]. The above sets of data, taken together, lend support for the potential utility of this PARP inhibitor in non-oncological diseases including ARDS.

With an eye for future repurposing of olaparib for the experimental therapy of ARDS, the objective of this study was to evaluate the effect of olaparib on inflammatory mediator production and cellular bioenergetics in mononuclear cells isolated from the peripheral blood of ARDS patients.

## Materials and methods

### Patients and PBMC preparation

Clinical studies were conducted in accordance with the Declaration of Helsinki and according to a clinical protocol approved by the cantonal authority of the Canton Vaud, Switzerland (2022–01193). Blood samples were collected from healthy volunteers and ARDS patients between May 2022 and February 2025. Eight healthy human volunteers aged 18–50 years, 62.5% women, and 8 ARDS patients aged 47–72 years, 25% women, were included. Blood collections were performed within 24 h of diagnosis (designated as "Day 1") and 7 days later (designated as "Day 8"). The inclusion criteria for the study were as follows: (a) Age: above18 years and under 85 years; (b) Sex: male & female; (c) ARDS with mechanical ventilation for less than 48 h; (d) Berlin criteria used for ARDS diagnosis; (e) Moderate to severe ARDS; (f) Mechanical ventilation used. The exclusion criteria were as follows: (a) Pregnancy; (b) A preexisting medical condition with a life expectancy less than 3 months; (c) Evidence of cardiogenic pulmonary edema; (d) Age under 18 years or above 85 years; (e) ARDS lasting for longer than 48 h; (f) ARDS of exclusively viral origin (without bacterial superinfection) and (g) Treatment with olaparib or another PARP inhibitor. With these criteria, 8 patients were included and followed over time, with clinical parameters recorded (Tables 1, 2 and 3).

**Table 1:** Patient characteristics and respiratory parameters at inclusion

	**Sex**	**Age**	**ARDS** **etiology**	**Microorganism**	**SOFA**	**Pa/O**_**2**_ (P/F) ratio *	PEEP **	MV duration ***
						mm Hg	cm H_2_O	h
Patient 1	M	72	COVID-19; bacterial superinfection	undetermined	6	86	14	41
Patient 2	M	57	Community-acquired pneumonia	undetermined	8	83	12	53
Patient 3	M	50	Aspiration pneumonia	*Klebsiella pneumoniae*	9	52	8	24
Patient 4	M	65	Influenza A; bacterial superinfection	undetermined	7	59	12	32
Patient 5	F	60	Influenza A; bacterial superinfection	undetermined	7	102	9	43
Patient 6	F	47	Primary pneumonia, tonsillitis	*Streptococcus pyogenes*	11	70	10	28
Patient 7	M	63	Primary pneumonia	undetermined	10	65	13	64
Patient 8	M	48	Influenza A; bacterial superinfection	undetermined	8	55	18	22

^*^ Lowest observed Pa/O_2_—ratio after intubation

^**^ Level of PEEP set at lowest Pa/O_2_

^***^ Duration of mechanical ventilation (MV) from intubation till 1 st blood sample collection

**Table 2:** Patient parameters at Day 8

	Still in ICU*	**SOFA**	Still on MV **	**Pa/O**_**2**_** (P/F) ratio**	**PEEP**
	days			mm Hg	cm H_2_O
Patient 1	NO	3	NO	230	0
Patient 2	NO	1	NO	233	0
Patient 3	NO	2	NO	308	0
Patient 4	YES	4	YES	246	14
Patient 5	YES	3	YES	201	10
Patient 6	YES	3	YES	159	7
Patient 7	YES	4	YES	238	8
Patient 8	YES	7	YES	242	18

^*^ For patients who have left ICU before day 8, the last available values are mentioned

^**^ Ongoing mechanical ventilation (MV) at improvement

**Table 3:** Patient parameters at improvement and outcome

	Improvement time*	**SOFA**	Still on MV**	**Pa/O**_**2**_** (P/F) ratio**	**PEEP**	**MV total duration**	**Left ICU** Day 30***
	days			mm Hg	cm H_2_O	days	
Patient 1	6	3	YES	221	7	8	YES
Patient 2	6	1	NO	209	1	5	YES
Patient 3	4	2	NO	308	0	4	YES
Patient 4	9	2	YES	309	8	11	YES
Patient 5	10	3	YES	258	8	28	NO
Patient 6	6	3	YES	251	8	14	YES
Patient 7	8	4	YES	228	8	13	YES
Patient 8	15	4	YES	285	7	15	YES

^*^ Duration from intubation till achievement of improvement criteria (spontaneous ventilation, PEEP < = 8 cm H_2_O, PaO_2_ > = 200 mm Hg)

^**^ Ongoing mechanical ventilation at improvement

^***^ All patients were alive on Day 30

PBMCs were prepared as described [38] by the Ficoll density gradient method (Ficoll-Paque plus) and suspended in RPMI 1640 medium (Sigma, St. Louis, MO, USA) supplemented with 10% fetal calf serum, 10 IU/ml penicillin, 10 μg/ml streptomycin (Gibco, Gaithersburg, MD, USA), and 2 mM L-glutamine (Sigma). Cell viability and count were determined with trypan blue using a hemocytometer. Olaparib was purchased from Sigma-Aldrich (St. Louis, MO, USA).

### Immunophenotyping

The expression of cell surface receptors was investigated to evaluate the cell population present in PBMCs. PBMCs (0.3 × 10^6^) were transferred to polystyrene tubes (BD Biosciences) and stained with the following monoclonal antibodies: anti-CD45-FITC, anti-CD3-APC, anti-CD14-BV711, anti-CD16-APC-Cy7, anti-CD66b-BV421 and 7AAD. All antibodies were from BD Biosciences. Samples were incubated with fluorochrome-conjugated monoclonal antibodies for 15 min in the dark at room temperature. Two milliliters of MACS-buffer (PBS, 0.1% BSA and 2 mM EDTA) were added into each tube and the samples were centrifuged at 800 g for 5 min at 4 °C. The cells were suspended in 500 μl of PBS paraformaldehyde 1% and were incubated in the dark on ice for 30 min. The samples were washed with 2 mL MACS-buffer, and the cells were suspended in 0.3 mL with MACS-buffer and analyzed by flow cytometry. Assays were performed by multiparameter flow cytometry using the LSRFORTESSA flow cytometer (BD Bioscience). Event acquisition was performed using FACSDiva software and analyses were performed using FlowJo software (BD Bioscience).

### Western blotting

PBMCs were centrifuged for 5 min at 400 × g to resuspend the pellet in RIPA Lysis and Extraction Buffer (Thermo Scientific, Waltham, MA, USA) supplemented with Halt™ Protease and Phosphatase Inhibitor Cocktail (Thermo Scientific) just before use. Protein concentrations were determined by measuring the absorbance in Pierce™ Coomassie Plus (Bradford) Assay Reagent (Thermo Scientific) using an Infinite 200 Pro plate reader (Tecan, Männedof, Switzerland). Samples were prepared for gel electrophoresis in Bolt™ LDS Sample Buffer (4x) (Invitrogen) and Bolt™ Reducing Agent (10x) (Invitrogen, Thermo Scientific) according to manufacturer’s instructions, loaded in Bolt™ 4%–12% Bis–Tris Plus Gels (Invitrogen, Thermo Scientific) and ran at constant 120 V. Proteins were transferred to PVDF (polyvinylidene difluoride) membranes by dry transfer using the iBlot™ 2 Device and Transfer Stacks (Invitrogen). Membranes were blocked with TBS/0.1% Tween/5% Milk Buffer (TBST/5% Milk). Protein expression was evaluated by Western blotting using specific antibodies in appropriate dilutions (see below). The incubation was done overnight at 4 °C under agitation, then the membranes were washed with TBST, and incubated for 1 h at room temperature with the secondary antibodies. The detection was performed with Amersham ECL™ Prime Western Blotting Detection Reagent (GE Healthcare, Pittsburgh, PA, USA) and the chemiluminescence was detected with Azure Imaging System 300 (Azure Biosystems, Dublin, CA, USA).

The following antibodies were used: anti-PARP-1 rabbit polyclonal antibody at 1:1,000 dilution (cat. no. 9542S; Cell Signaling Technology, Beverly, MA, USA); anti-poly (ADP-ribose) monoclonal antibody (10H) at 1:500 dilution (ALX-804–220; Enzo Life Sciences, Inc., NY, USA); β-actin mouse monoclonal antibody (cat. no. 47778; Santa Cruz Biotechnology, Dallas, TX, USA) used as loading control at 1:5,000 dilution. The secondary anti-rabbit IgG, HRP-linked antibody purchased from Cell Signaling Technology (cat. no. 7074S) and was used at 1:10,000 dilution. The secondary goat anti-mouse IgG-HRP (sc-2005; Santa Cruz Biotechnology, Dallas, TX, USA) was used at 1:250,000 dilution.

### Cytokine measurements

For patients and controls, 10 ml of blood was collected in EDTA tubes. Blood samples were centrifuged to separate plasma, which was frozen in aliquots at − 80 °C until use. To supernatant, PBMCs (1 × 10^6^/ml) were incubated in nonadherent tubes in a 5% CO_2_ incubator at 37^◦^C in the presence of 100 ng/ml LPS (Escherichia coli; O127:B8). Olaparib (10 μM) or its vehicle was added 1 h before LPS. After incubation, the tubes were gently vortexed to homogenize the cell suspension and centrifuged at 400 × g for 10 min at 4 ^◦^C. The supernatant was stored at − 80 ^◦^C until use. Plasma and supernatant samples were later analyzed to quantify IL-6 (#558,276), IL-8 (#558,277), IL-10 (#558,274), MCP-1 (#558,287), MIP-1α (#558,325), TNF-α (#558,273), IL-17A (#560,383), IL-1β (#558,279), IFN-γ (#558,269), and soluble ICAM-1 (sICAM-1, human CD54; #560,269). Cytokine measurements were performed using Cytometric Bead Array (CBA) Flex Set kits (BD Biosciences). Data acquisition was done on a BD LSRFortessa flow cytometer with FACSDiva software, and data were analyzed using FCAP Array v3.0 software (BD Biosciences).

### Determination of cellular bioenergetics – seahorse assay

The Seahorse XFe24 flux analyzer (Agilent Technologies, Santa Clara, CA, USA) was used to estimate cellular bioenergetics of PBMC. PBMCs (1 × 10^6^ cells/ml) were incubated in nonadherent tubes in a 5% CO_2_ incubator at 37 °C, for 4 h with olaparib (10 µM) or its vehicle. H_2_O_2_ (100 µM, (Sigma-Aldrich) was then added and the cells were further incubated for 2 h. Next, the samples were washed with 2 ml of cold PBS and centrifuged at 400* g* for 10 min at room temperature. The supernatant was discarded, and the cells were placed in 500 µl Seahorse medium at 37ºC and 100 µL/well (i.e. 0.2 × 10^6^ cells) were transferred to the Seahorse plate. Four wells per condition were used as technical replicates and the obtained 4 values were averaged during data analysis. The plate was centrifuged, at 200 g for 1 min and incubated in a non-CO_2_ incubator at 37 °C for 30 min. Next, each well on the plate received 400 µl Seahorse medium at 37ºC and the Extracellular Flux Analysis was conducted as described [50]. The O_2_ consumption rate (OCR) after oligomycin (1 µM) was used to estimate the ATP production rate. Moreover, carbonyl cyanide-4-trifluoromethoxy phenylhydrazone (FCCP, 1.5 µM) was used to estimate the maximal mitochondrial respiratory capacity. Electron flux through complex III and I was blocked, respectively, with antimycin A (0.5 µM) and rotenone (0.5 µM). Residual activity in the presence of these inhibitors was considered non-mitochondrial OCR. All data were normalized with total protein content, using the BCA protein assay (Thermo Scientific).

### Statistical analysis

Power analysis was performed as follows: a two-group comparison was planned to detect a difference between a control mean of 1.0 and a treatment mean of 0.5, assuming both groups have a standard deviation of 0.3. The design involved two independent groups of equal size, a two-sided significance level of 0.05, and a desired power of 0.80. The analysis was based on a standard two-sample t-test under normal approximation. Using these parameters, the required sample size per group was calculated according to the formula that relates the difference between means, the assumed standard deviation, and the combined critical values for the desired alpha and beta levels. Substituting the specified values yields approximately 6 participants per group. To allow for potential dropouts or technical failures, blood was collected and PBMCs were isolated from a total of 16 subjects (8 healthy controls and 8 ARDS patients). Sample sizes vary by assay due to sample availability; exact n for each experiment is provided in the figure legends.

For comparisons between two groups, a paired Student's t-test was used (when appropriate, e.g., baseline vs treatment in the same individuals). For comparisons among more than two groups, one-way ANOVA was performed with Sidak’s post-hoc test for multiple comparisons.

To evaluate whether clinical severity was associated with the magnitude of the olaparib response, we performed correlation analyses between Sequential Organ Failure Assessment (SOFA) scores and the percent effect of olaparib. Associations were first assessed using both Pearson’s correlation coefficient (r), to test for linear relationships, and Spearman’s rank correlation coefficient (ρ), to assess monotonic relationships independent of distributional assumptions. Because samples were obtained at two different time points (Day 1 and Day 8), we additionally examined whether the relationship between SOFA score and olaparib effect was influenced by sampling day. Stratified correlation analyses were therefore conducted within each time point separately. Furthermore, an analysis of covariance (ANCOVA) framework was applied to account for the potential confounding effect of time point, testing SOFA score as a continuous predictor while including day as a categorical covariate.

All statistical analyses were performed using GraphPad Prism 8.0 (GraphPad Software) and two-sided p values < 0.05 were considered statistically significant. Data are expressed as mean ± standard error of the mean (SEM). Statistical significance was defined as *p* < 0.05 and **p < 0.01.

## Results

### Characterization of leukocyte subpopulations in PBMCs

**Fig. 1:**
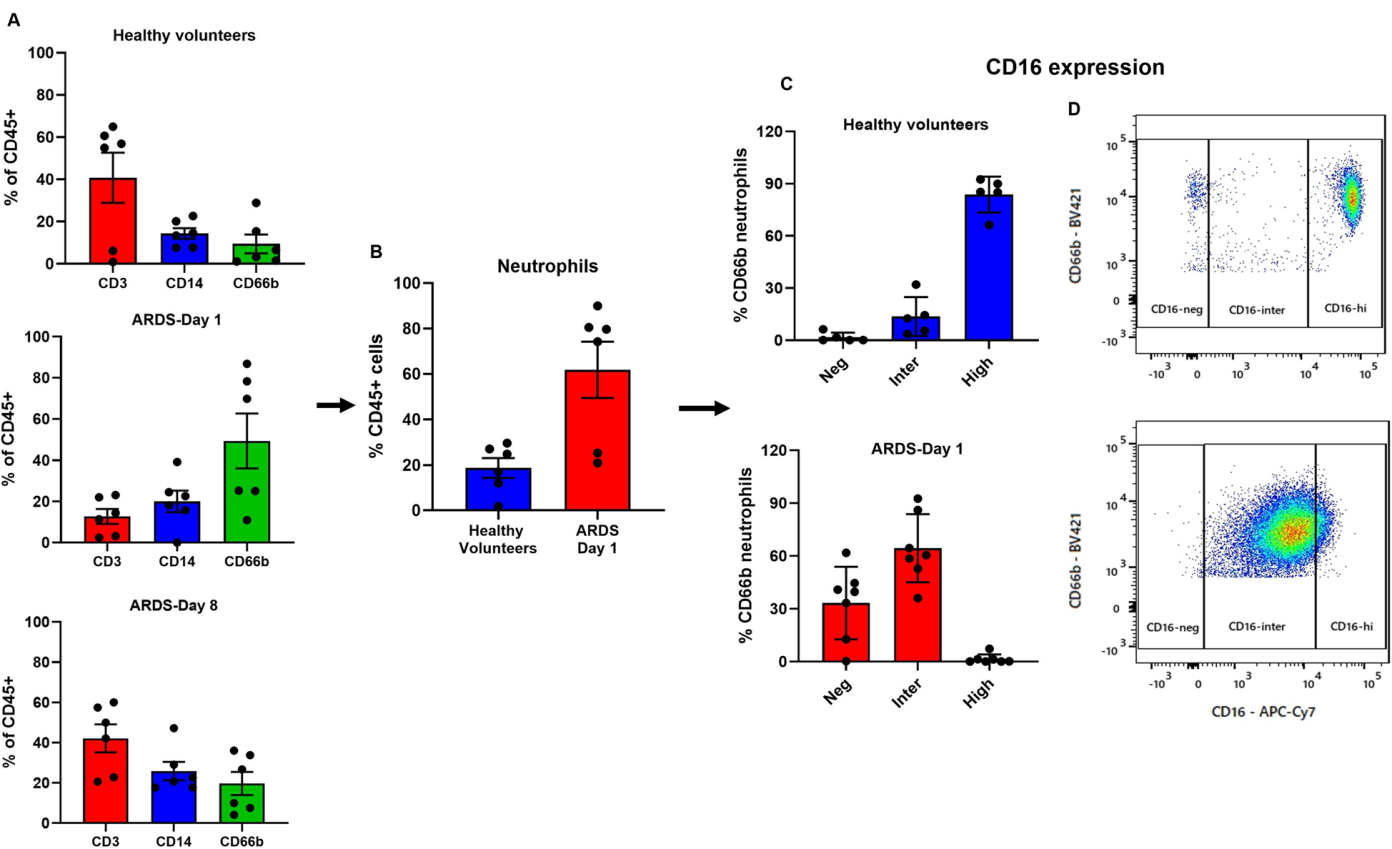
Expression of surface receptors in PBMCs from healthy volunteers (HV) and ARDS patients (Day 1). **A** PBMCs (0.3 × 10^6^) from HV (*N* = 7) and ARDS Day 1 patients (*N* = 6) were stained to define leukocyte subpopulations: lymphocytes (CD3), monocytes (CD14), and neutrophils (CD66b). **B** Low-density neutrophils in PBMCs from HV and ARDS Day 1 were identified; % CD66b^+^ neutrophils gated on viable CD45^+^ cells. **C** Distribution of CD16 expression (negative, intermediate, high) in CD66b^+^ neutrophils. **D** Representative dot plots of CD16 populations in CD66b^+^ neutrophils from one HV (top) and one ARDS Day 1 patient (bottom). Data are shown as individual values with mean ± SEM

PBMCs obtained from healthy volunteers exhibited a leukocyte population with a predominance of lymphocytes (~ 48%), whereas in ARDS patients on Day 1 the most abundant cells were neutrophils (~ 49%). By Day 8, the leukocyte profile of ARDS patients became comparable to that of healthy volunteers, with lymphocytes again predominant (~ 42%) (Fig. 1A). The neutrophil population in control patients presented with a high expression of CD16, while the neutrophils of ARDS patients on Day 1 presented with a predominantly intermediate expression of CD16 (Fig. 1B-D).

### Western blotting analysis

**Fig. 2:**
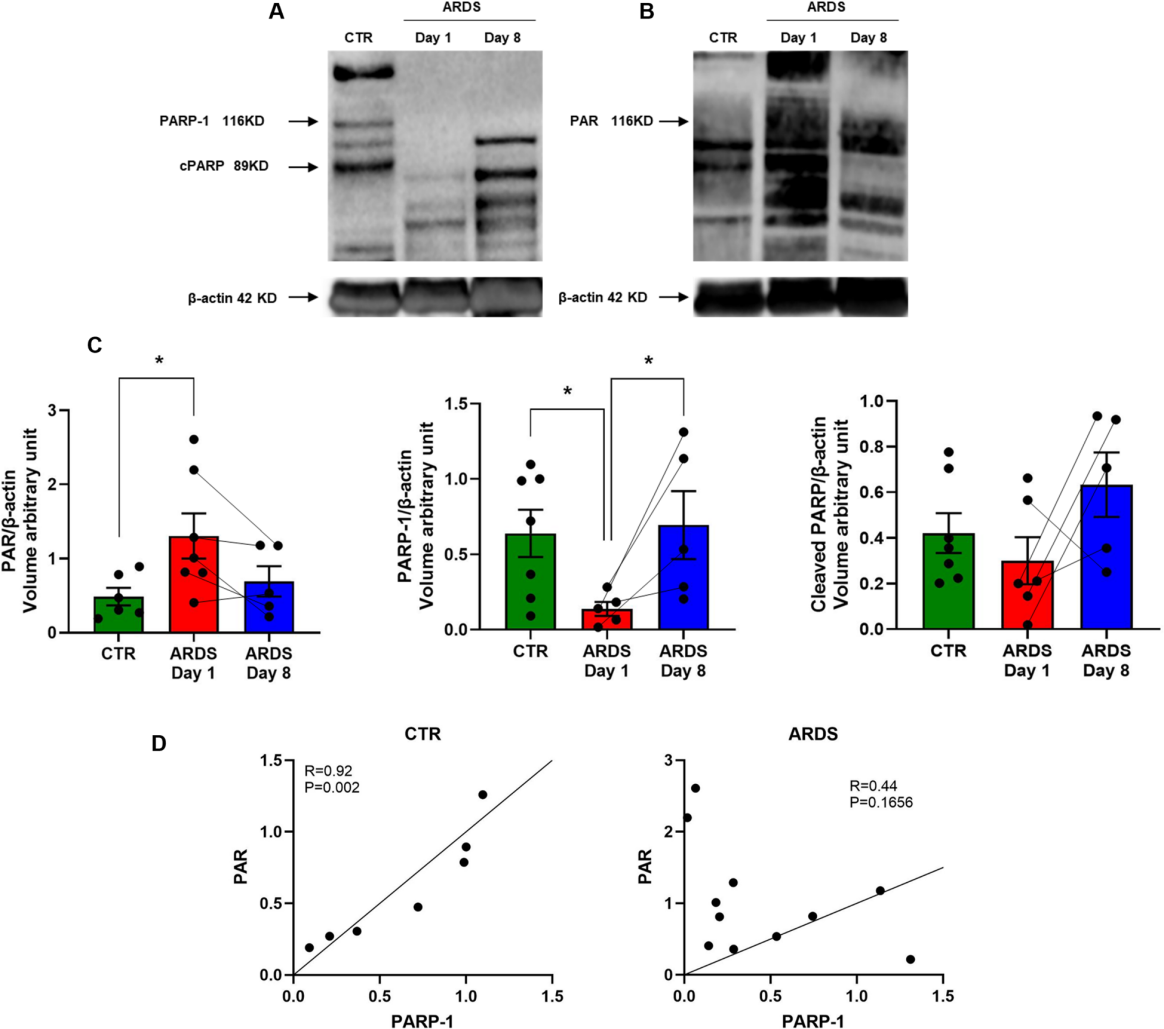
Detection of PARP-1, cPARP-1, and the presence of protein-bound PAR polymers (PARylation) in PBMCs by Western blotting. Expression of PARP-1 and protein PARylation (PAR) was evaluated in human PBMC (1 × 10^6^ cells) from healthy volunteers (control, CTR) and ARDS patients (Day 1 and Day 8). Bands represent protein expression of (**A**) PARP-1 and cPARP-1, **B** PARylation. **C** Densitometric analyses of PAR, PARP-1, and cPARP-1, normalized to β-actin as the loading control; data points that belong to the same patient on Day 1 and Day 8 are connected with a dotted line. **D** Pearson correlation test between PAR and PARP-1 in CTR (*N* = 7) and ARDS patients (Day 1 and Day 8 samples pooled (*N* = 12). Data are individual values with mean ± SEM. *p < 0.05; ns, not significant

PARP-1, cPARP, and PAR polymers were all detected in the analyzed PBMC samples from healthy volunteers and ARDS patients. In the control samples, full-length PARP-1, cleaved PARP (cPARP) and PAR polymers were detectable, with PARP predominantly present in its full-length physiological form, and to a smaller extent in the cleaved form –– with the cleaved form representing 39 ± 9% of the PARP protein detected (Fig. 2A-C). In PBMCs from ARDS patients on Day 1, PARylated proteins were approximately 2-times more abundant than in the PBMCs of control subjects, while PARP-1 was less abundant and the cleaved form representing the majority (72 ± 10%) of the protein detected (Fig. 2A-C). On Day 8 of ARDS, PARylation was lower than what was detected in Day 1 (and was comparable to the degree of PARylation detected in healthy control samples), and PARP was present both in cleaved and uncleaved forms, with the cleaved form representing 46 ± 14% of the protein detected.

In healthy control samples, a strong correlation was observed between the presence of PARP-1 expression and PARylation (R = 0.92 and *P* = 0.002). However, no correlation between PARP and PAR was apparent in the PBMCs of the ARDS patients (Fig. 2D).

### Cytokine measurements

**Fig. 3:**
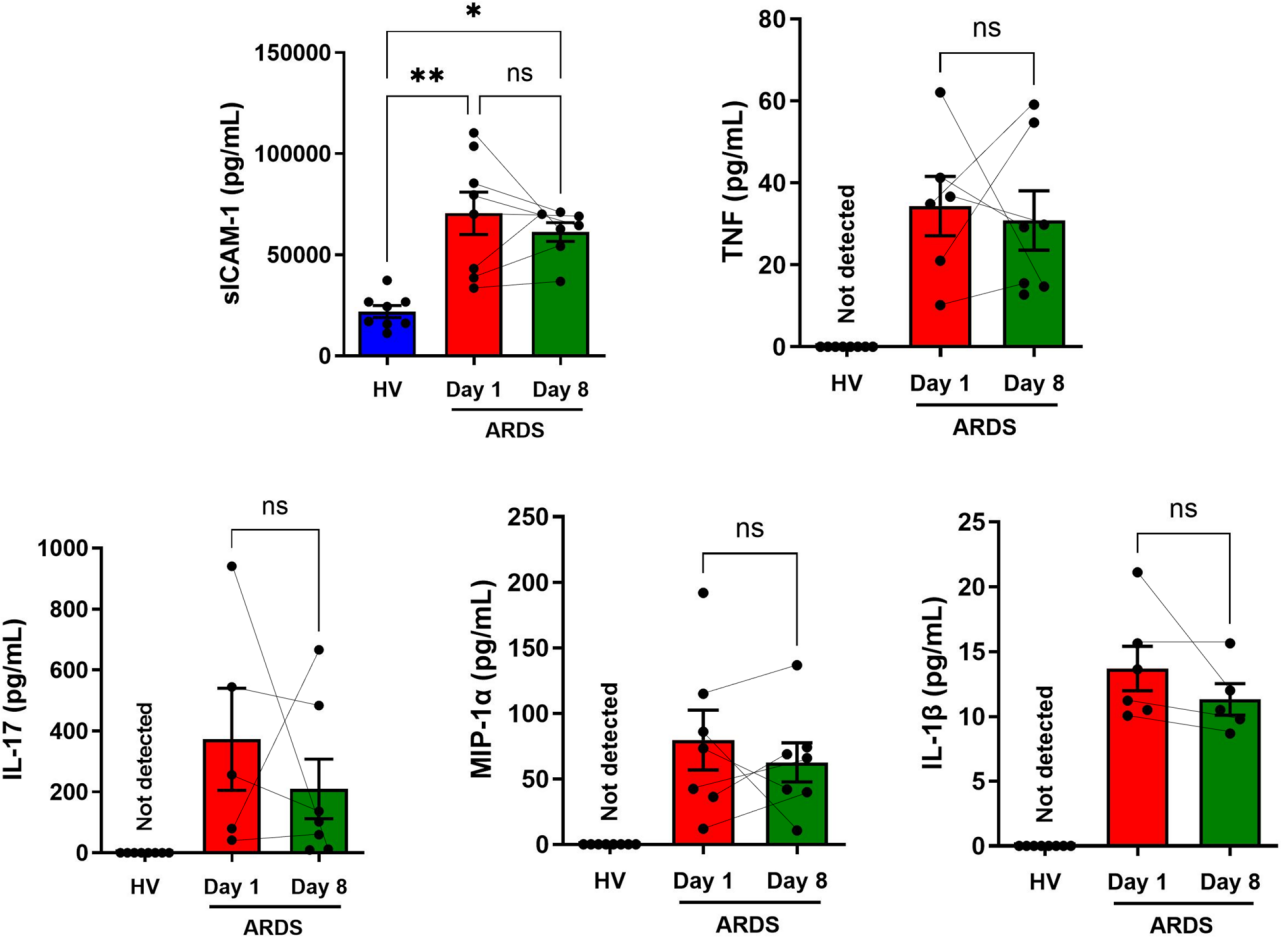
Quantification of baseline cytokine plasma levels. Whole blood was collected in EDTA tubes and centrifuged for separation of plasma, which was frozen in aliquots at − 80 °C until use. Quantification of IL-6, IL-8, IL-10, MCP-1, MIP1-α, TNF-α, IL-17A, IL1-β, IFN-γ and sICAM-1 were performed by the CBA assay in healthy volunteers (*N* = 8) and ARDS patients on Day 1 (*N* = 6) and Day 8 (*N* = 7). Data are individual values with mean ± SEM. *p < 0.05, **p < 0.01; ns, not significant

**Fig. 4:**
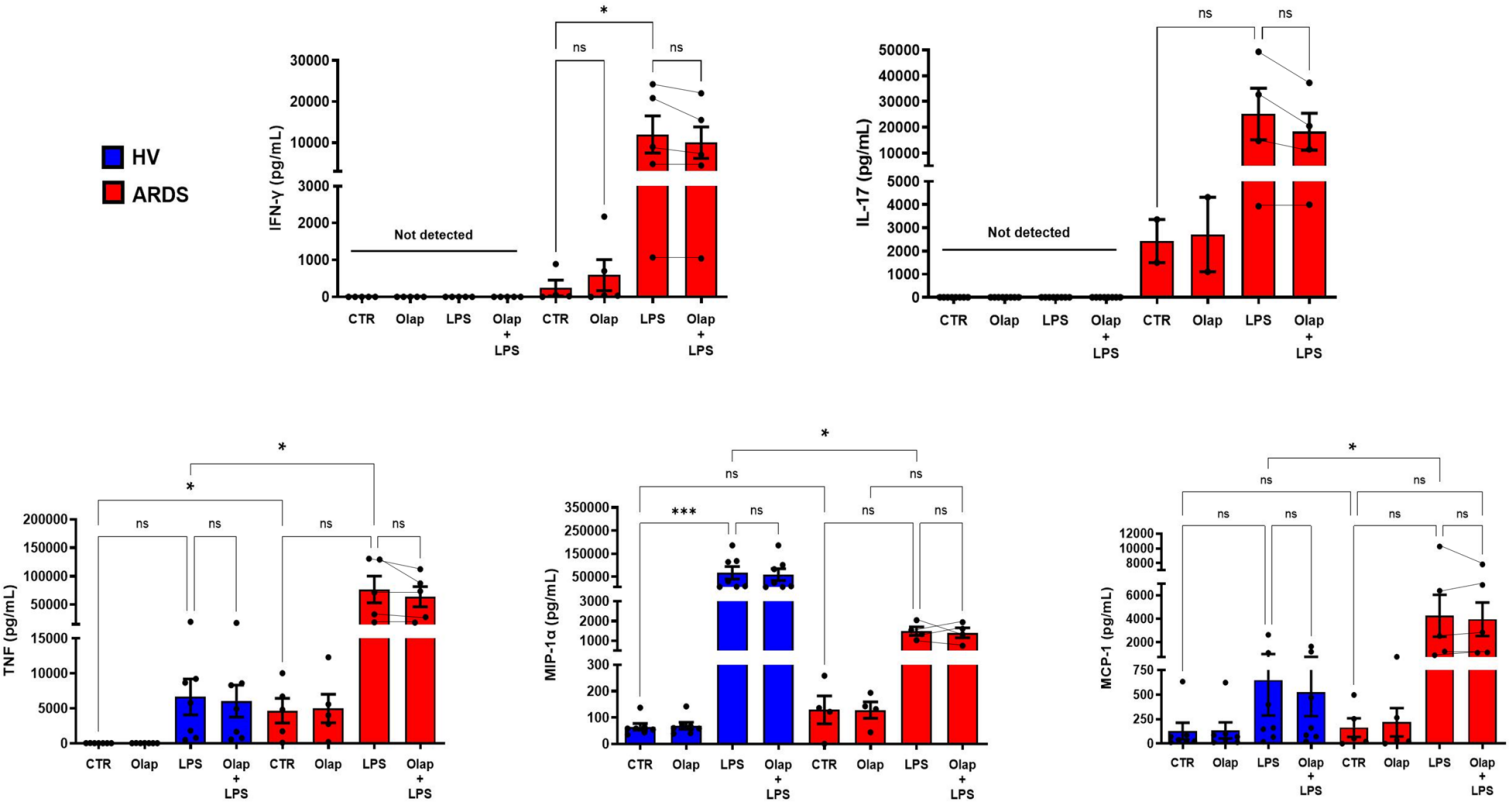
Effect of olaparib on cytokine secretion in human PBMCs from healthy volunteers and ARDS patients. PBMC (0.5 × 10^6^ cells) from healthy volunteers (*N* = 7) or Day 1 ARDS patients (*N* = 5) were incubated for 4 h with or without LPS (100 ng/ml). Cultures were untreated, or olaparib 10 µM was added 1 h before exposure to LPS. Culture supernatants were collected at 5 h. Quantification of IL-6, IL-8, IL-10, MCP-1, MIP1-α, TNF-α, IL-17A, IL1-β, IFN-γ and sICAM-1 were performed using a CBA assay. Data are individual values with mean ± SEM. *p < 0.05; **p < 0.01; ***p < 0.001; ns, not significant

TNF-α, sICAM-1, IL-17, MIP-1α and IL-1β were all detectable in the plasma of ARDS patients while in healthy volunteers only extremely low levels of MCP-1 and sICAM-1 were detected (Fig. 3). After LPS stimulation, PBMCs from healthy controls and ARDS patients both produced cytokine and chemokine responses. PBMCs isolated from the blood of ARDS patients exhibited an enhanced LPS-induced IFN-g, TNF-α and MCP-1 response compared to the control cells' response to LPS, while the MIP-1α generation was lower in the ARDS PBMCs than in the control PBMCs (Fig. 4). Pretreatment with olaparib did not have any significant effect on these responses, although for the TNF-α and IL-17 responses, trends (approximately 15% decreases) were noted (*p* = 0.19 and *p* = 0.11, respectively) (Fig. 4).

### Bioenergetic analysis

**Fig. 5:**
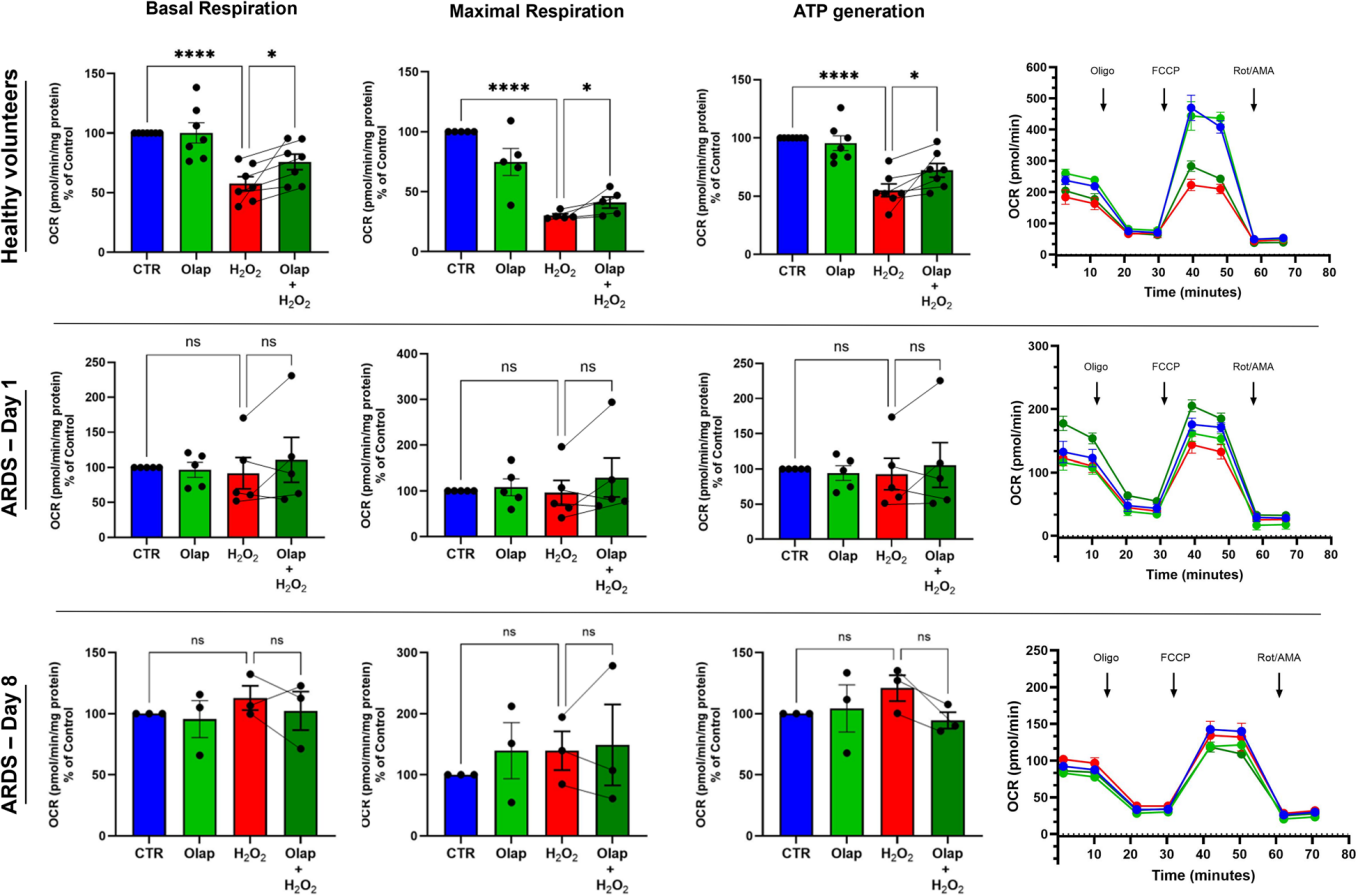
Effect of olaparib on cellular bioenergetics of PBMC subjected to oxidative stress by H_2_O_2_. PBMC (0.2 × 10^6^ cells) from healthy volunteers (*n* = 7), ARDS, Day 1 (*n* = 5) and ARDS, Day 8 (*n* = 3), were incubated for 4 h with olaparib (10 µM) followed addition of H_2_O_2_ 100 µM in the last 2 h. **A** Seahorse bioenergetics profile (average) OCR before and after administration of various pharmacological agents. Injections of oligomycin (Oligo), FCCP and rotenone + antimycin A (Rot/AMA) are shown with arrows. **B** Basal respiration, **C** Maximal respiratory capacity and (**D**) ATP generation. Data were normalized to total protein and are shown as mean ± SEM; within-subject initial OCR was scaled to 100%. Absolute basal OCR (pmol O₂·min⁻1·mg⁻1 protein): HV 238 ± 14 (*N* = 7), Day 1 83 ± 17 (*N* = 5), Day 8 87 ± 3 (*N* = 3). After FCCP: HV 409 ± 20, Day 1 176 ± 10, Day 8 142 ± 4; both ARDS groups vs. HV, *p* < 0.01. *p < 0.05; ns, not significant

**Fig. 6:**
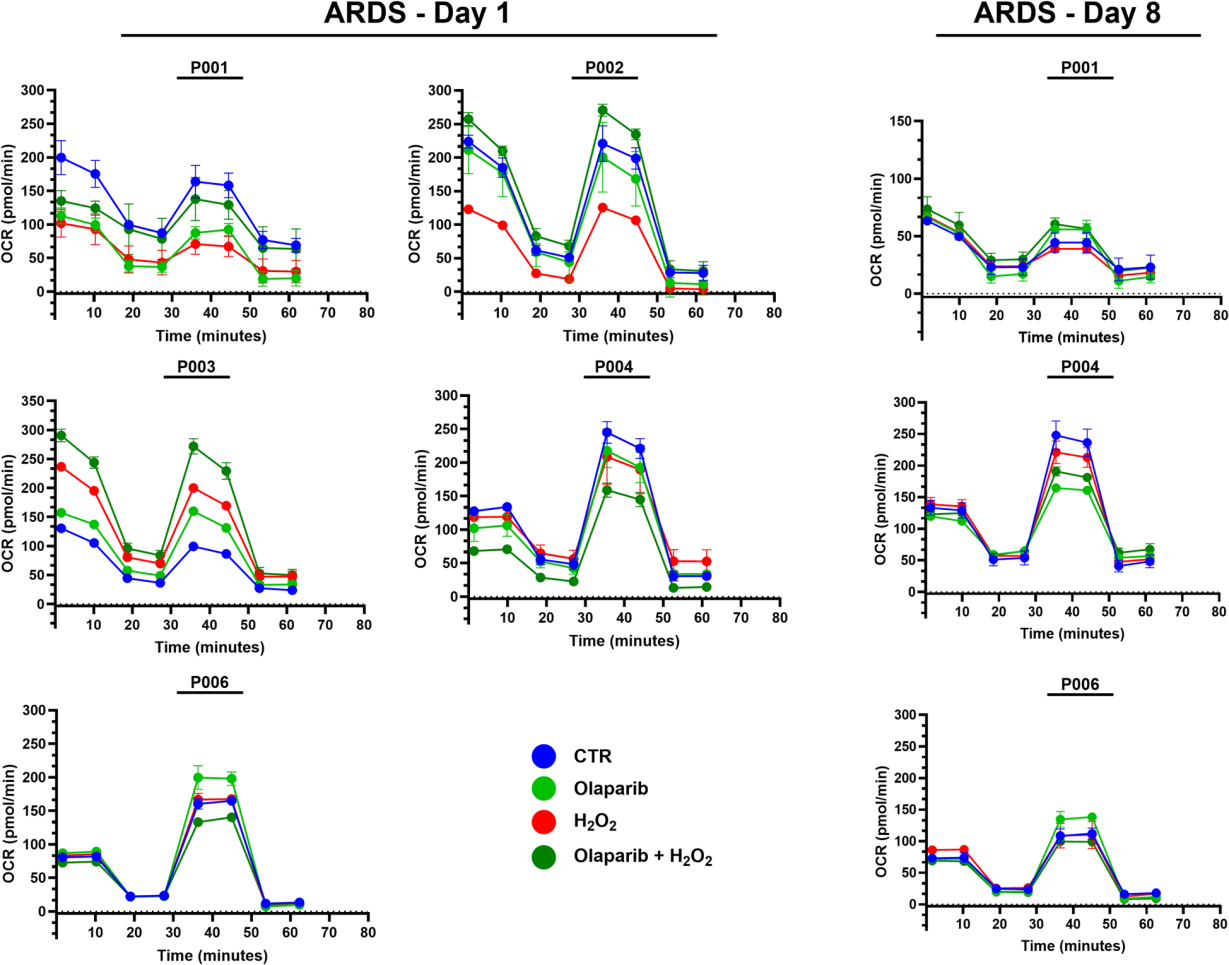
Effect of olaparib on cellular bioenergetics of PBMCs subjected to oxidative stress by H_2_O_2_. Individual OCR tracings from ARDS Day 1 (*N* = 5) and Day 8 (*N* = 3) patients are shown. Injections of oligomycin (Oligo), FCCP and rotenone + antimycin A (Rot/AMA) are shown with arrows. Data represent mean ± SEM of 4 technical replicates from each Seahorse run

In PBMCs from healthy controls, oxidative stress suppressed the various cellular bioenergetic parameters, and this effect was slightly, but significantly attenuated by olaparib pretreatment (Fig. 5). In contrast, in PBMCs isolated from ARDS patients –– which exhibited a significant baseline suppression of cellular bioenergetic parameters –– exposure to oxidative stress in vitro did not produce any significant further metabolic suppression, and the PARP inhibitor was without any significant functional effect when the data were analyzed as a group aggregate (Fig. 5). Analysis of individual patient responses revealed highly heterogeneous patterns with respect to the cells' response to oxidative stress and to the effect of olaparib (Fig. 6).

### Exploratory correlation analyses of clinical and biochemical parameters

To further characterize relationships among clinical variables and PARP-related readouts, we performed various exploratory correlation analyses using the available ARDS samples. At disease onset (Day 1), no statistically significant associations were observed between normalized PARP activation (PAR levels) or PARP cleavage and clinical severity indices, including SOFA score and P/F ratio (all *p* > 0.1). PARP activation showed a moderate positive correlation with SOFA score (r = 0.71, *p* = 0.12). No consistent associations were observed between PARP-related measures and P/F ratio.

Because ARDS samples are enriched in neutrophils (Fig. 1), which have been reported to express lower levels of PARP-1 than lymphocytes (see: Discussion), we examined whether neutrophil proportion could account for the observed PARP-related changes. Within the ARDS cohort (D1 and D8 combined), neutrophil proportion did not significantly correlate with normalized PARP expression (Pearson r = –0.52, *p* = 0.16) or normalized PARP activity (*r* = 0.44, *p* = 0.24). To further address potential compositional confounding, we performed analysis of covariance (ANCOVA) including disease group (ARDS vs. healthy controls) and neutrophil proportion as a covariate. In these models, neutrophil proportion did not significantly predict PARP activation (β = 0.0097, *p* = 0.27), while its association with PARP expression was modest and borderline (β = –0.0094, *p* = 0.049). Importantly, sensitivity analyses restricting the comparison to healthy controls and Day 1 samples, or modeling controls, Day 1, and Day 8 as separate groups, yielded similar results, with neutrophil proportion failing to show a robust or consistent effect. Together, these analyses indicate that neutrophil enrichment does markedly influence the observed alterations in PARP activity.

Given the broad age range of the study population, we additionally examined whether age was associated with neutrophil proportion, PARP-related biochemical readouts, or PARP activity. Within the ARDS cohort, age showed an inverse correlation with neutrophil proportion (*r* = –0.51, *p* = 0.089), whereas no such association was observed in healthy controls (*r* = –0.08, *p* = 0.89). However, age did not significantly correlate with normalized PARP expression, PARP activity (PAR levels), or PARP cleavage in ARDS samples (all *p* > 0.3). When age and disease group were included together in regression models, disease status — but not age — was the dominant determinant of neutrophil enrichment. Overall, these analyses indicate that age does not significantly influence PARylation or other PARP-related biochemical parameters in this dataset, and that the observed alterations are primarily associated with disease status rather than age.

Strong correlations were observed among PARP pathway–related biochemical measures. As already shown in Fig. 2D, in healthy control PBMCs, PARP-1 expression strongly correlated with PARylation levels, indicating tight coupling between enzyme abundance and catalytic activity. In contrast, no correlation between PARP-1 expression and PARylation was observed in ARDS samples. PARP expression showed no significant correlation with cleaved PARP in healthy controls, showed a strong correlation in Day 1 ARDS samples (*r* = 0.97, *p* = 0.001), but showed no correlation in Day 8 ARDS samples (*r* = 0.44, *p* = 0.56). Together, these findings indicate the cleavage (and inactivation) of PARP-1 during the early phase of ARDS.

Considering the marked heterogeneity of the cellular bioenergetic responses observed in PBMCs from ARDS patients (Fig. 6), we further explored whether disease severity might influence the response of PBMCs to olaparib ex vivo. PBMCs from Day 1 ARDS patients exhibited higher variability in their responses to olaparib or H_2_O_2_ than PBMCs from Day 8 (variability in maximal OCR values in D1 vs. D8 samples: 110 ± 23 vs. 31 ± 13 pmol/min, *n* = 5/n = 3, *p* < 0.05). In the Day 1 subset of ARDS patients (*n* = 5), the % effect of olaparib on the OCR of the PBMCs showed a strong positive association with the patients' SOFA score, with a Pearson correlation of r = 0.779 (*p* = 0.120) and a Spearman correlation of ρ = 0.900 (*p* = 0.037). In this cohort, there were two patients (P003 and P006) where olaparib increased the PBMC's bioenergetic responses (Fig. 6), and these two patients had the two highest SOFA scores at the time of enrolment (Table 1). Thus, more severe disease may predict a better functional response to the PARP inhibitor – perhaps due to more pronounced PARP activation in these cells, and despite a concomitant, significant degree of PARP1 cleavage (Fig. 2c). In Day 8 PBMCs, correlation analysis was not feasible due to the small cohort size (*n* = 3). However, we have noted that in 2 out of the 3 Day 8 patients' PBMCs (P001, P006), olaparib tended to slightly improve basal mitochondrial respiration rates (Fig. 6). While these observations are based on a limited number of patient samples, it is conceivable that disease severity and disease stage may influence the respiratory rate of the PBMCs as well as their bioenergetic responses to PARP inhibition.

## Discussion

Based on the extensive prior evidence that PARP inhibitors (including olaparib) exert cytoprotective and anti-inflammatory effects in various non-oncological disease models (see: Introduction), in the present study we assessed the effects of olaparib on LPS-induced inflammatory mediator production and oxidative stress–induced bioenergetic alterations in PBMCs from healthy volunteers and ARDS patients. We have selected a clinically relevant concentration of olaparib (10 µM), which does not adversely affect human lymphocyte viability [38].

Our results show that PARP-1 protein is predominantly in its physiological, full-length form in PBMC from healthy volunteers, and catalyzes a relatively low level of baseline PARylation. In contrast, in the PBMCs of ARDS patients, PARP-1 expression is lower than control on Day 1, and the cleaved form of PARP becomes the predominant form. In addition, there is a markedly higher abundance of PARylated proteins on Day 1 in the PBMCs of ARDS patients than the PARylation observed in control PBMCs. This increased PARylation response was no longer apparent on Day 8 of ARDS. In control PBMCs –– but not in PMBCs from the ARDS patients –– a good correlation was observed between PARP-1 expression and PARylation. The partial cleavage of PARP is well characterized as a component of various forms of cell injury and cell death [8, 12, 46]. There is also good evidence that auto-PARylation of PARP decreases the enzyme's catalytic activity [45]. Thus, the findings presented in the current report are consistent with the following working hypothesis: At the early stage of ARDS, inflammatory mediators and oxidative stress trigger the activation of PARP in circulating cells. In fact, a similar activation of PARP has been previously observed in PBMCs from patients with myocardial infarction and diabetes mellitus [15, 43, 49]. In turn, PARP in ARDS PBMCs undergoes a proteolytic cleavage, perhaps as a response of the cell to prevent cell necrosis that PARP overactivation is known to elicit [7, 28, 40] . Indeed, PARP cleavage in human PBMCs has also been observed previously in various diseases, for instance in systemic lupus erythematosus [36]. Moreover, the catalytic activity of any remaining full-length PARP may be inhibited by auto-PARylation. The combination of auto-PARylation and PARP-1 cleavage is then the likely reason why olaparib loses its functional effect on cellular bioenergetics in PBMCs isolated from many Day 1 ARDS patients (see below).

Consistent with a systemic inflammatory response, ARDS patients had elevated cytokine levels in their plasma. PBMCs from both healthy individuals and ARDS patients mounted the expected cytokine responses upon LPS stimulation ex vivo –– although the degree of the cytokine production was, in some cases, different in PBMCs from healthy volunteers vs. in PBMCs from ARDS patients. Several prior studies have shown that the NF-kB pathway can be inhibited by pharmacological PARP-1 inhibition, culminating in the inhibition of the production cytokines such as TNF-1 and of chemokines such as MIP-1α [14, 27, 32, 34, 44]. In contrast to these findings, in the current experiments no significant cytokine modulation was observed after treatment with olaparib at the clinically relevant concentration used (10 µM). Our previous findings demonstrated that cytokine modulation in PBMCs is statistically significant only at a high (100 µM) and clinically irrelevant concentration of this PARP inhibitor [38]. It should be emphasized that 10 µM olaparib is sufficient to completely suppress PARP1-mediated PARylation responses [1, 10, 20, 22], thus, at concentrations higher than 10 µM, the effects of olaparib may include additional (perhaps non-specific actions.

As expected, olaparib had no significant effect on the basal bioenergetics of healthy PBMCs, and exerted protective effects against the oxidative stress-induced bioenergetic suppression in PBMCs isolated from the blood of healthy volunteers. These protective effects of the PARP inhibitor are likely related to the maintenance of the cellular levels of NAD^+^ and ATP in these cells [9]. Compared to the bioenergetic parameters of the PBMCs from healthy volunteers, PBMCs from ARDS patients exhibited significantly lower basal bioenergetic parameters, most likely due to the fact that these cells have already experienced pro-inflammatory/oxidant stress in vivo. Challenge of these cells with H_2_O_2_ in vitro did not produce any consistent further suppression of cellular bioenergetic parameters. Moreover, treatment of PBMCs from ARDS patients with olaparib –– with or without the oxidative stress challenge –– failed to influence the cellular bioenergetic parameters, when groups (Day 1 ARDS PBMCs or Day 8 ARDS PBMCs) were analyzed as a whole. However, correlation analysis and evaluation of individual patient responses indicated that in some PBMCs (e.g. those from patients from more severe ARDS on Day 1), olaparib produced a partial improvement of the mitochondrial function. We attribute the lack of consistent olaparib effect in PBMCs from ARDS either to the fact that the majority of PARP-1 in these cells is cleaved and/or inactivated, or perhaps to the fact that the cells are already at an advanced stage of injury/dysfunction/cell death process at which point modulation of PARP-mediated processes can no longer exert significant functional effects.

This study has several limitations. First, the sample size was small and only two time points were examined. Second, the healthy volunteer and ARDS patient groups were not completely identical in terms of age or sex. Third, the PBMC compositions differed between healthy controls and ARDS patients: healthy volunteers’ PBMCs were lymphocyte-predominant, whereas ARDS patient PBMCs contained a high proportion of neutrophils (especially on Day 1). The neutrophils present in ARDS PBMC preparations were likely low-density neutrophils, which have immunosuppressive effects such as reducing T, B, and NK cell proliferation and activity, thereby potentially modulating the immune response [29, 48]. Importantly, although PARP is constitutively present in most mammalian cell types, neutrophils represent an exception: PARP enzyme expression in neutrophils is low [5, 11, 13, 16]. However, our correlation analysis suggests that differences in cell population and PARP-1 expression did not have any major influence on our findings.

In conclusion, at clinically relevant concentrations, olaparib mitigated oxidant-induced bioenergetic decline in PBMCs from healthy individuals, while the PARP inhibitor had no significant overall effect on cytokine production or bioenergetic parameters in PBMCs from ARDS patients. Nevertheless, the analysis of individual responses to olaparib suggested partial responses in the PBMCs of some of the patients studied. We speculate that the lack of consistent effect of olaparib may be due to the fact that the timing of the intervention (ex vivo application of olaparib to isolated PBMCs) may have been introduced too late relative to the natural course of the disease, where PARP activation, auto-PARylation and consequent inactivation, as well as cleavage of the enzyme have already occurred.

The use of *ex vivo* PBMC PARylation responses has been used, with success, in some oncology studies to assess the functional effect of PARP inhibition [10, 23, 25]. Therefore, a similar approach may also be considered as a sentinel marker of PARP activity in critically ill patients. However, this application has significant limitations, because of the significant degree of PARP cleavage, as demonstrated in the current report.

Finally, it should be noted that the results presented in the current article do not fundamentally challenge the overall rationale of repurposing PARP inhibitors in non-oncological diseases [4], because PARP-1, the intended target cells in those conditions – in cardiomyocytes in myocardial infarction or neurons in neurodegenerative disease – is not subject to similar cleavage and therefore may remain responsive to pharmacological PARP inhibition.

## Supplementary Information

Supplementary Material 1.

## Data Availability

All data will be made available to qualified investigators upon reasonable request.
