# Supplementary material for: Cleavage and inactivation of poly(ADP-ribose) polymerase in peripheral blood mononuclear cells of patients with acute respiratory distress syndrome
Source: Respir Res. 2026 Mar 16;27:178. doi: 10.1186/s12931-026-03623-4 (PMC13104195; doi:10.1186/s12931-026-03623-4)

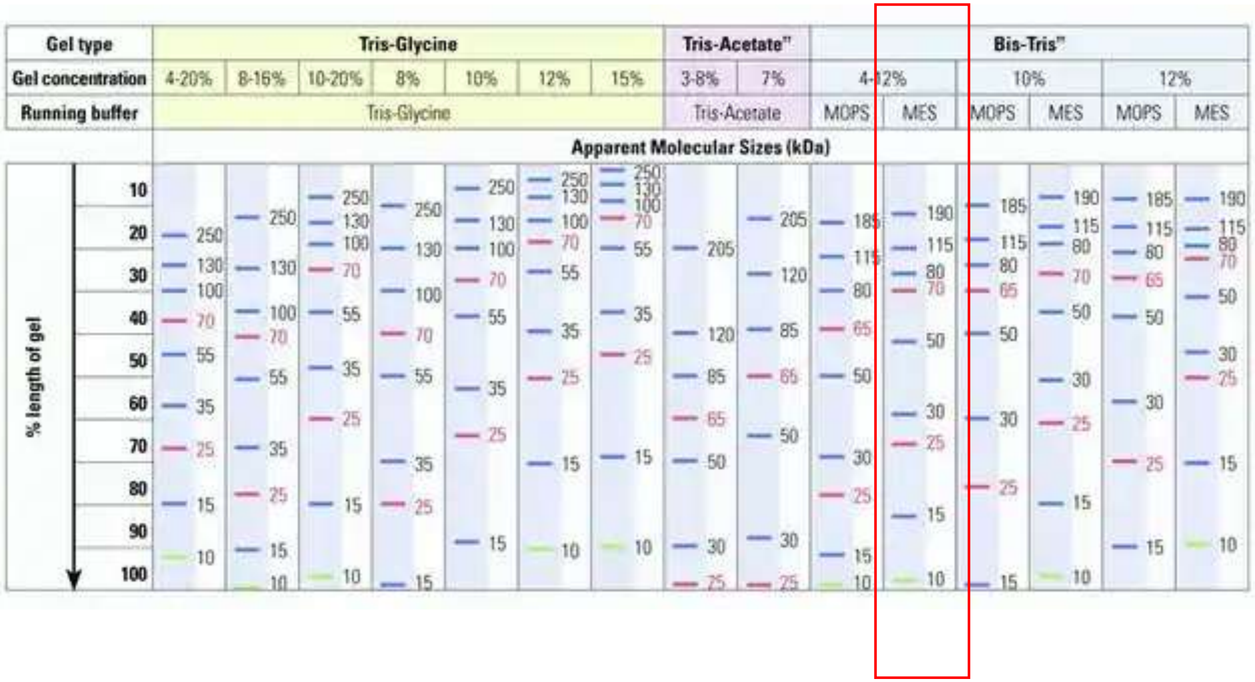

Gel type
Tris-Glycine
Tris-Acetate
Bis-Tris
Gel concentration
4-20% 8-16% 10-20% 8% 10% 12% 15%
3-8% 7%
4-12% 10% 12%
Running buffer
Tris-Glycine
Tris-Acetate
MOPS MES MOPS MES MOPS MES
Apparent Molecular Sizes (kDa)
% length of gel
10 20 30 40 50 60 70 80 90 100

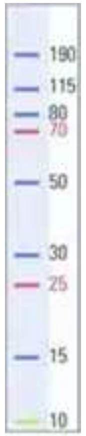

190
115
80
70
50
30
25
15
10

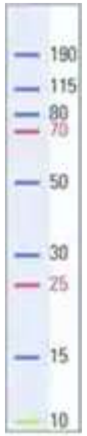

PageRuler Plus Prestained Protein Ladder

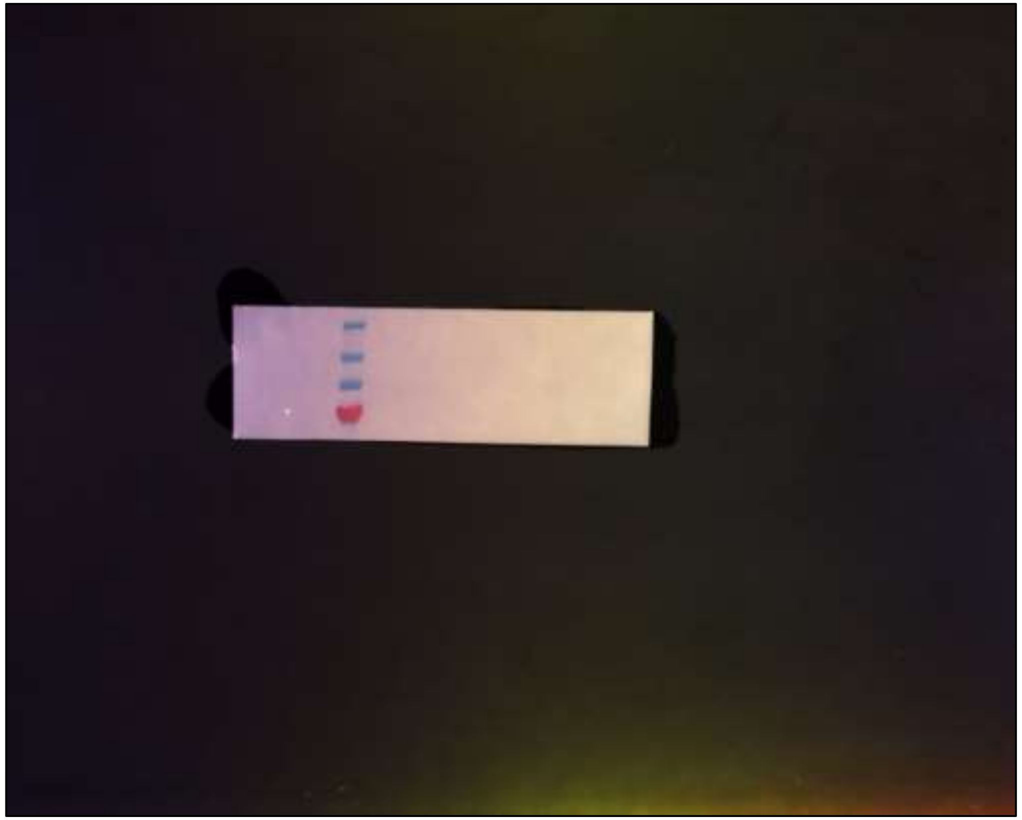

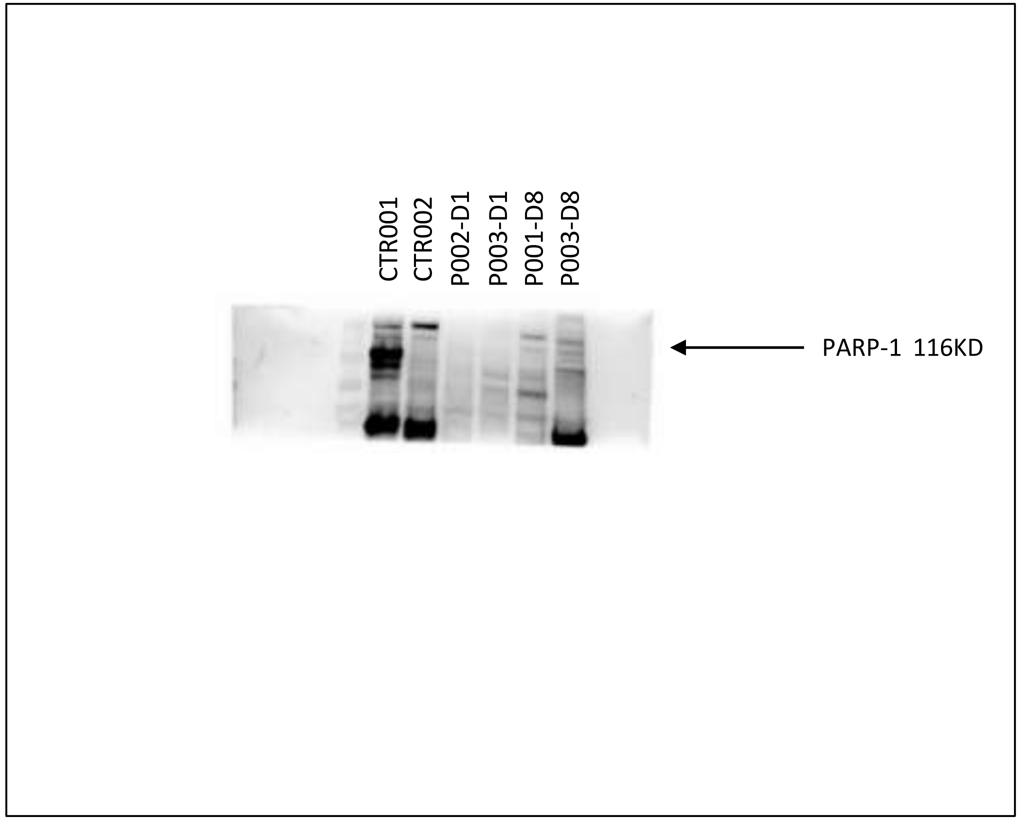

PageRuler Plus Prestained Protein Ladder

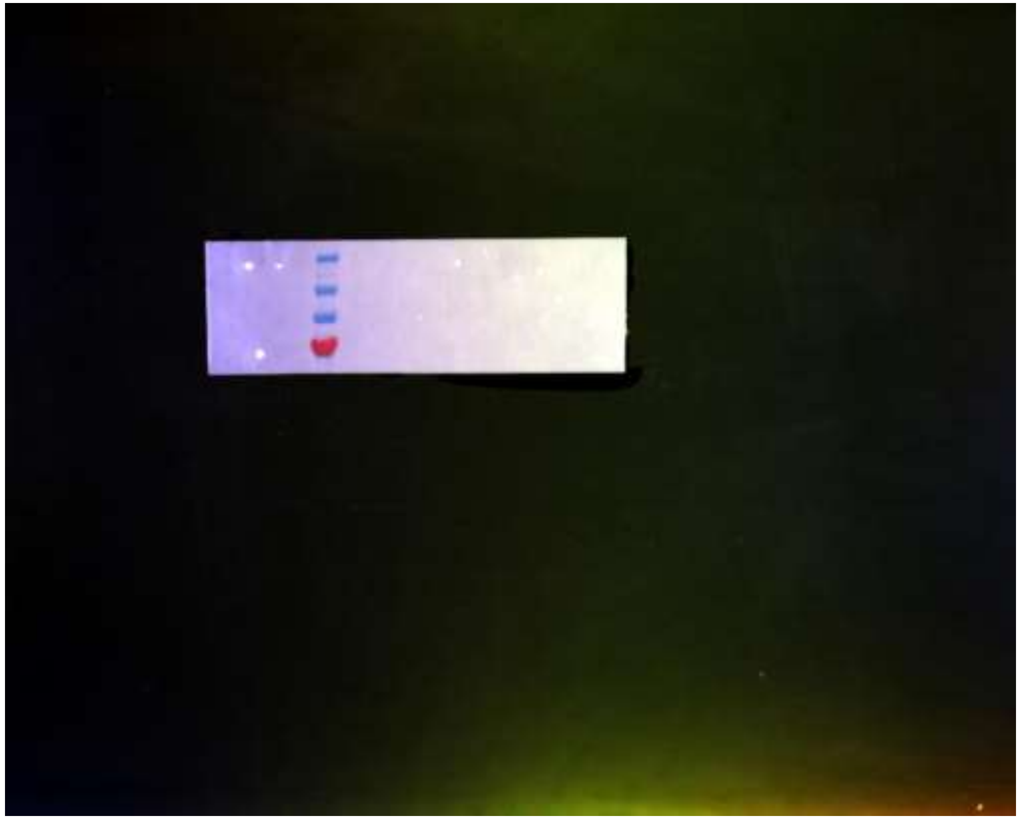

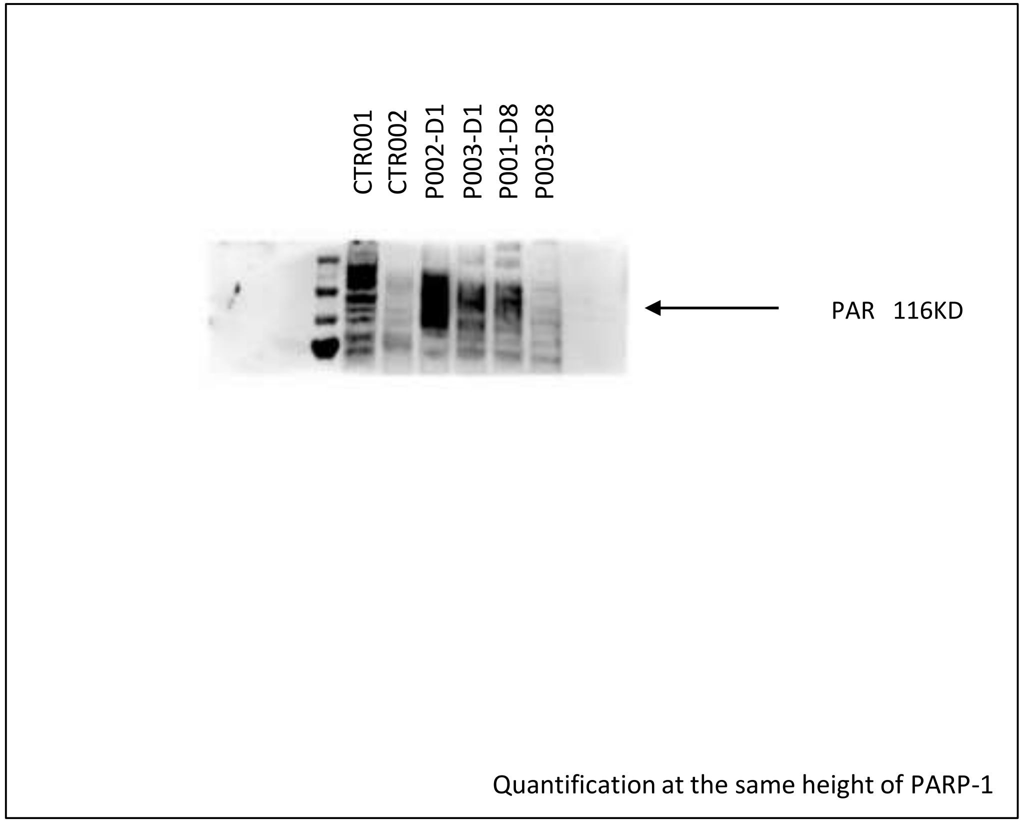

PageRuler Plus Prestained Protein Ladder

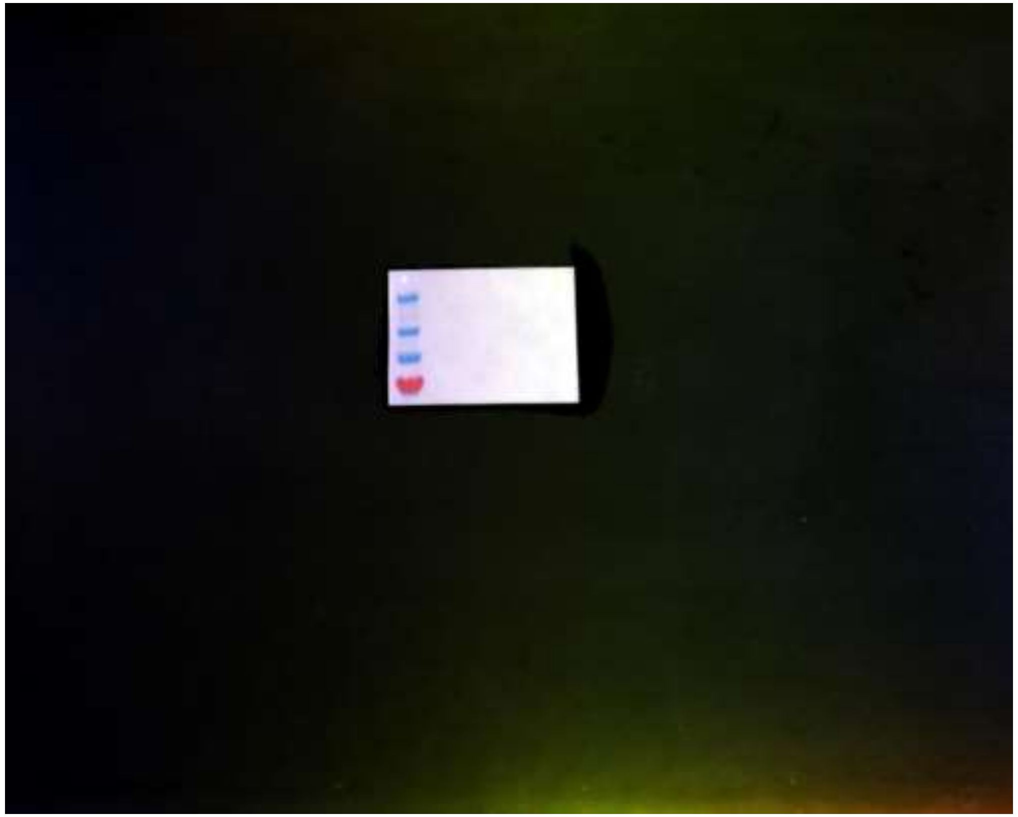

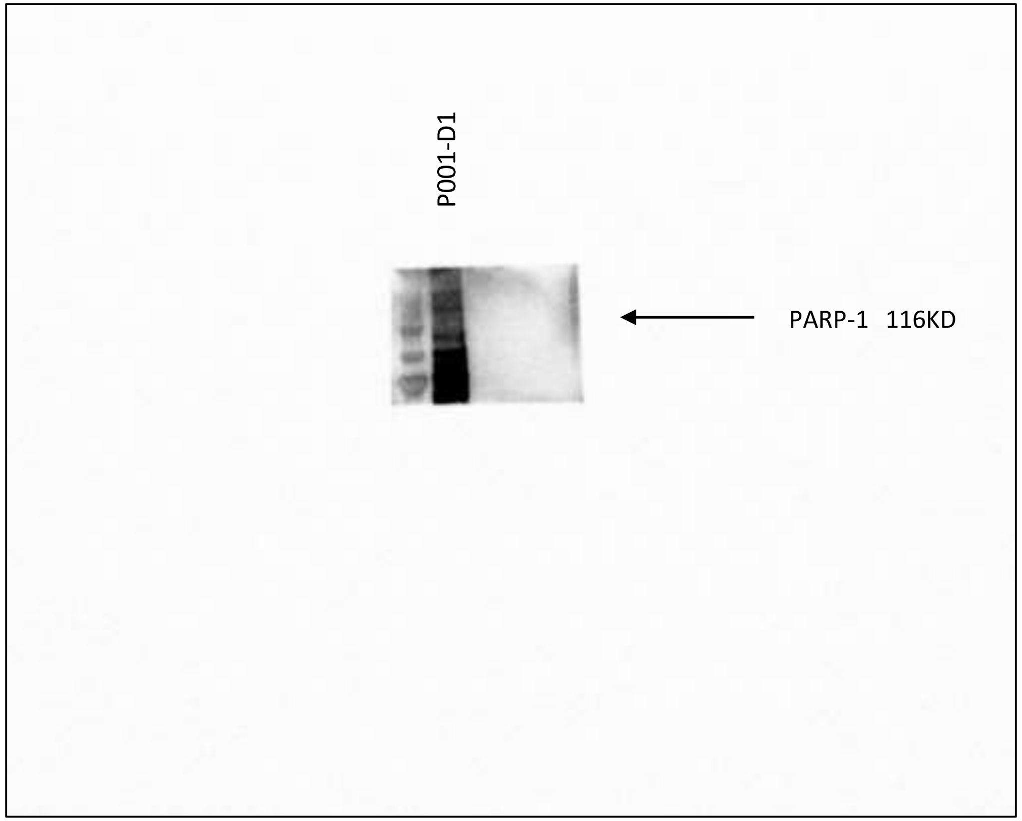

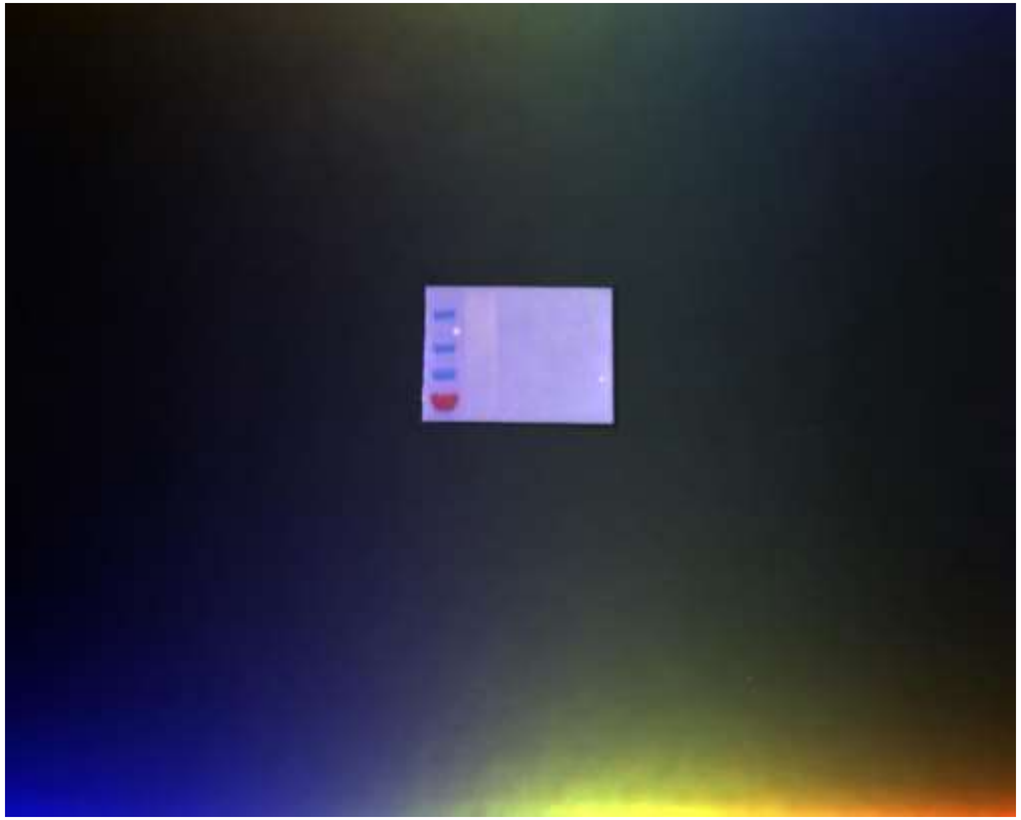

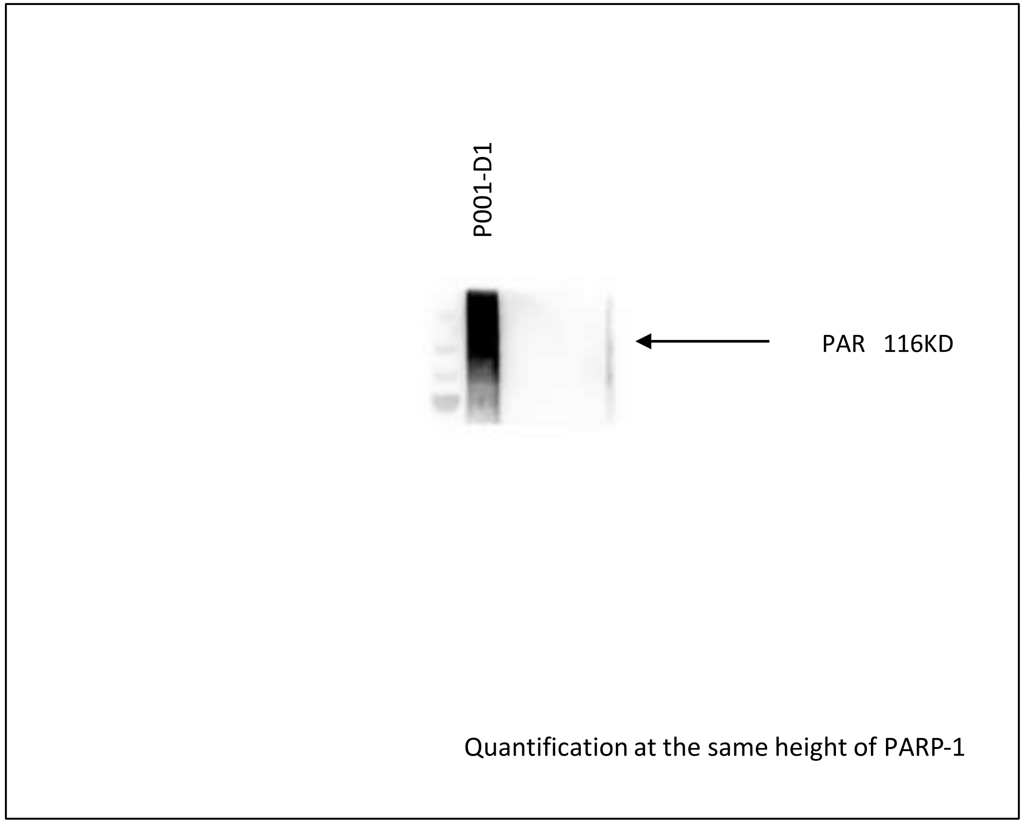
P001-D1
PAR 116KD
Quantification at the same height of PARP-1

PageRuler Plus Prestained Protein Ladder

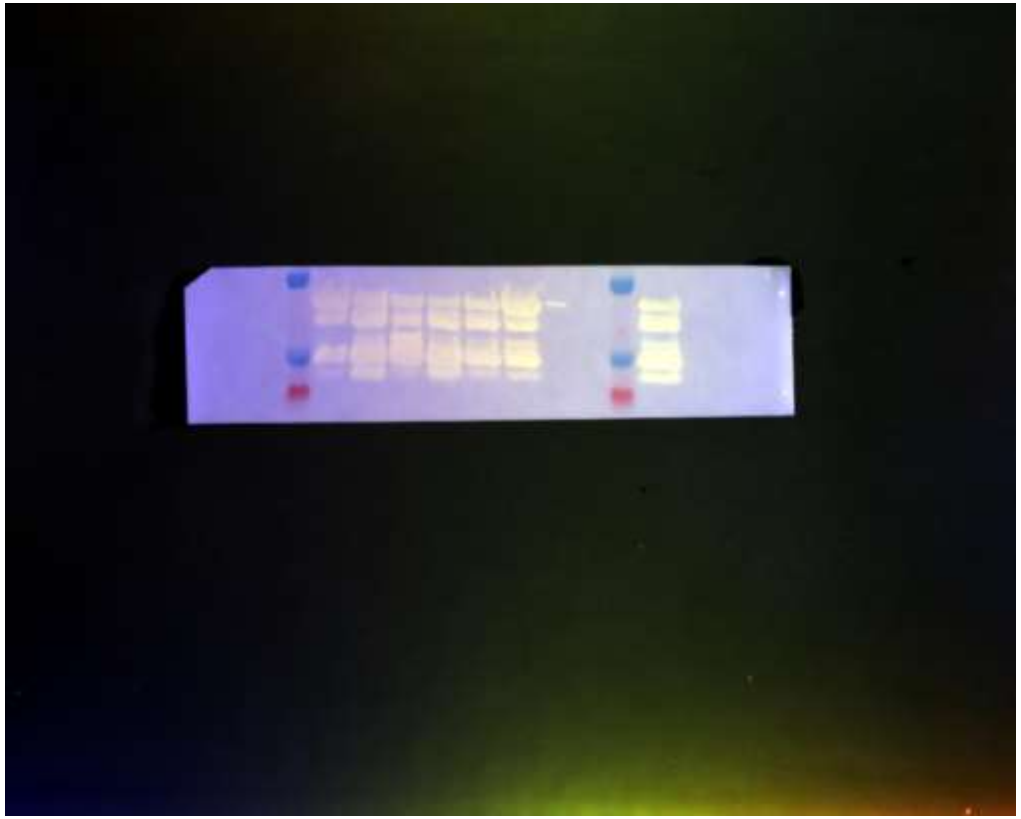

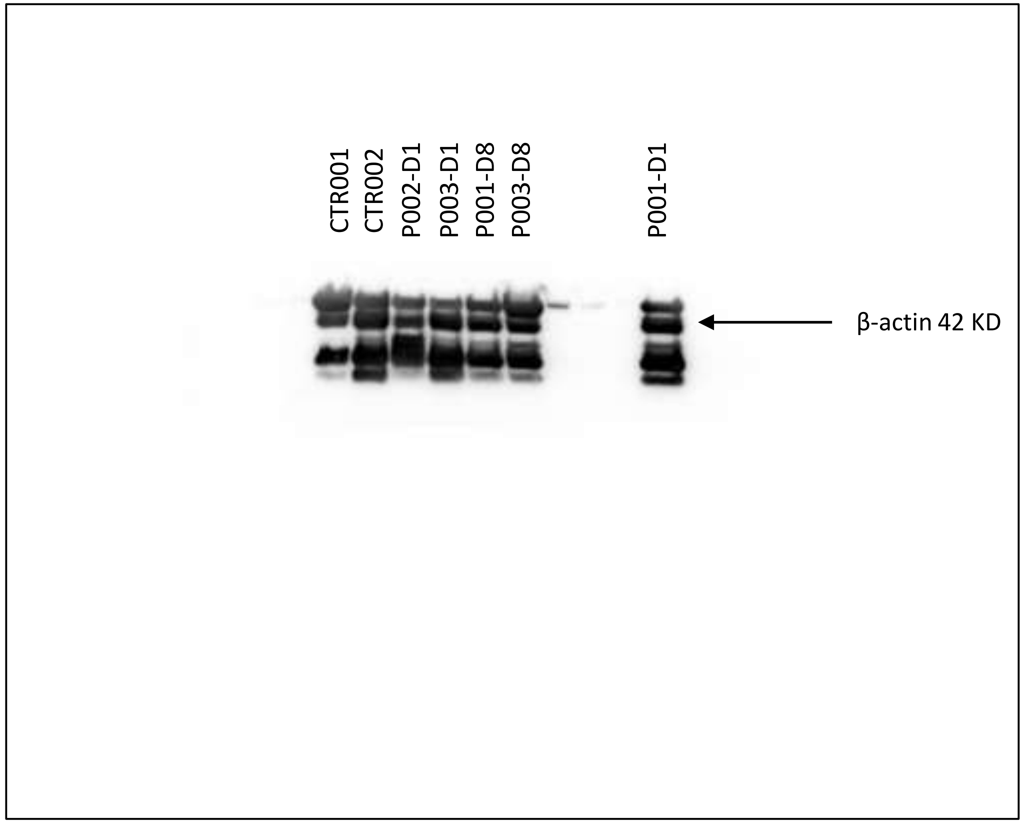

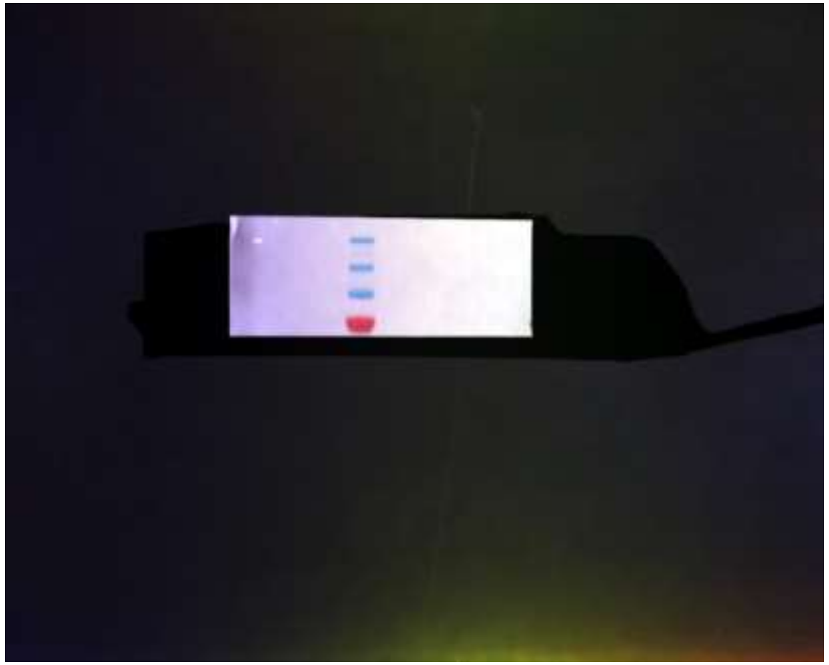

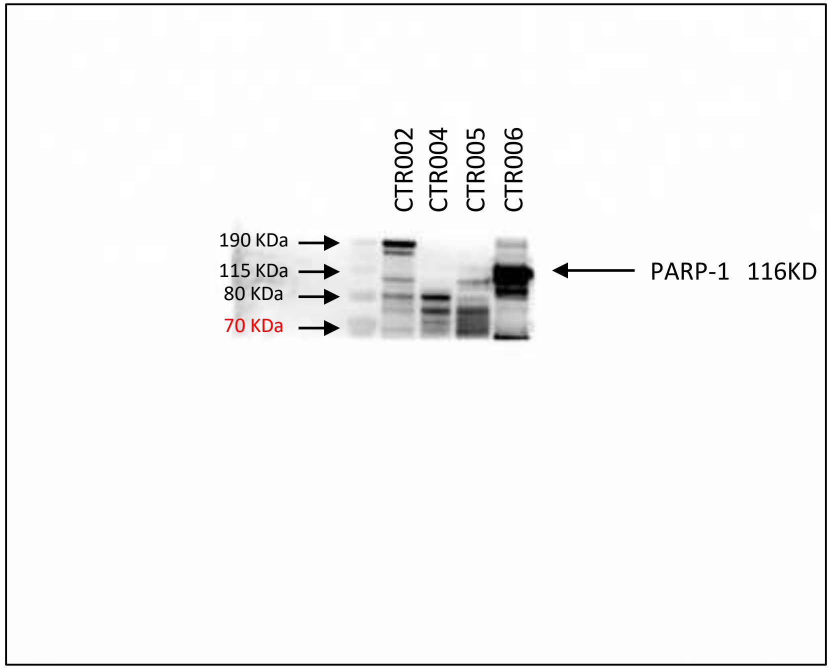

CTR002
CTR004
CTR005
CTR006
190 KDa
115 KDa
80 KDa
70 KDa
PARP-1 116KD

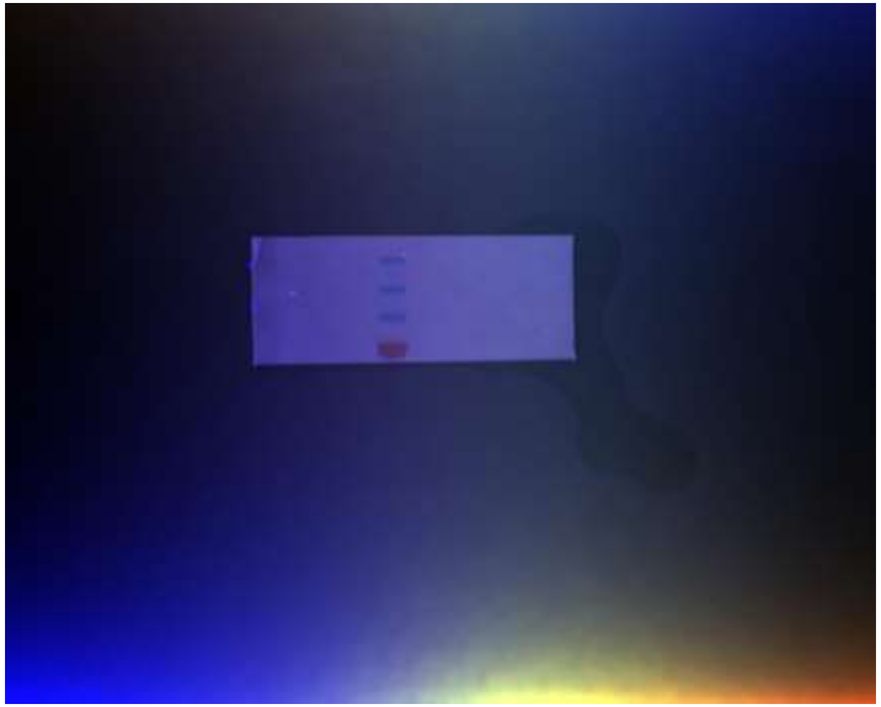

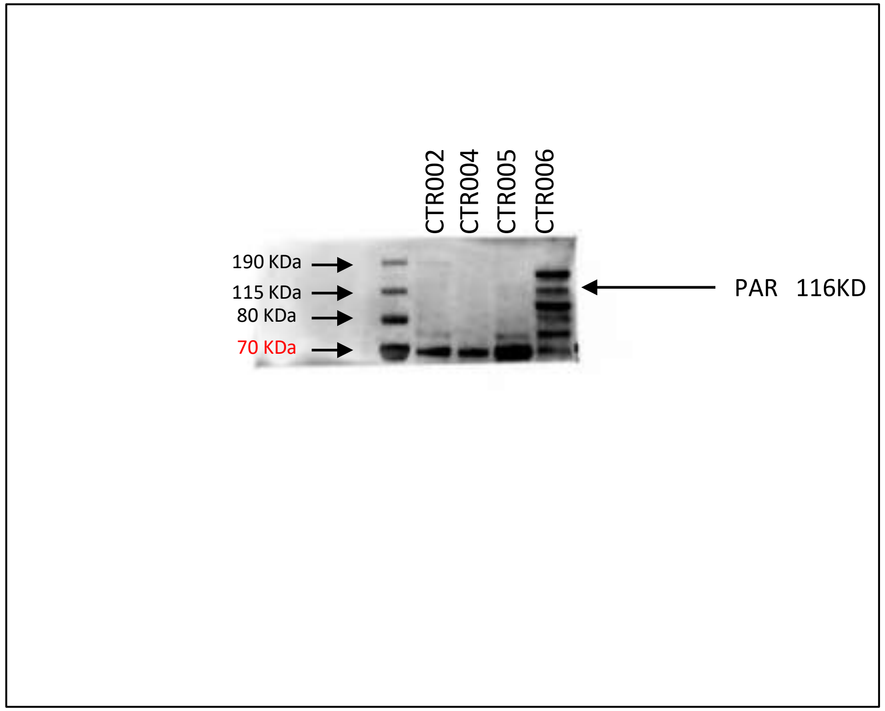
CTR002
CTR004
CTR005
CTR006
190 KDa
115 KDa
80 KDa
70 KDa
PAR 116KD

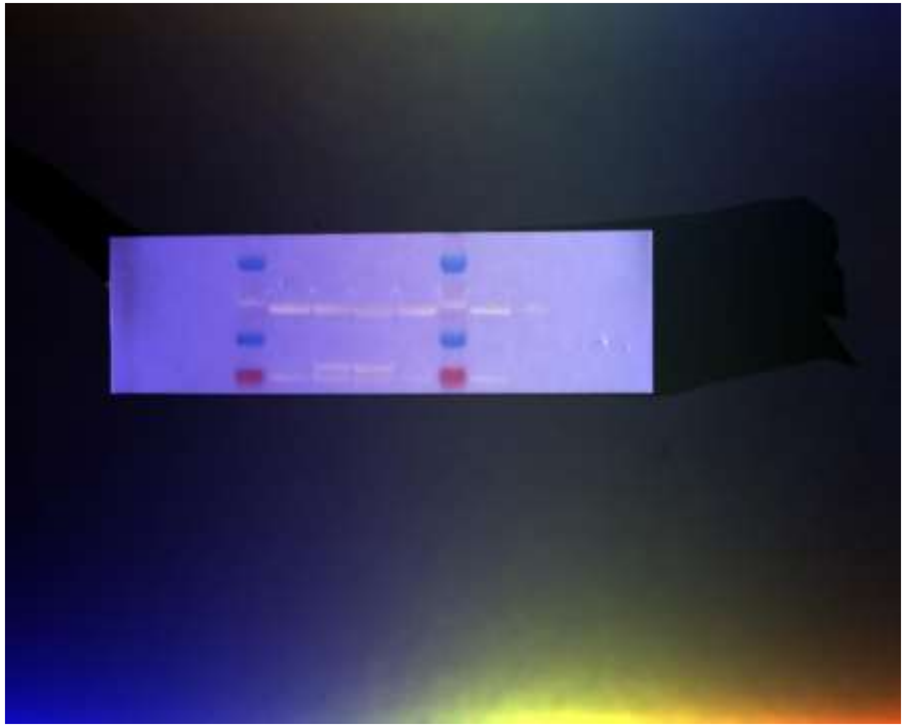

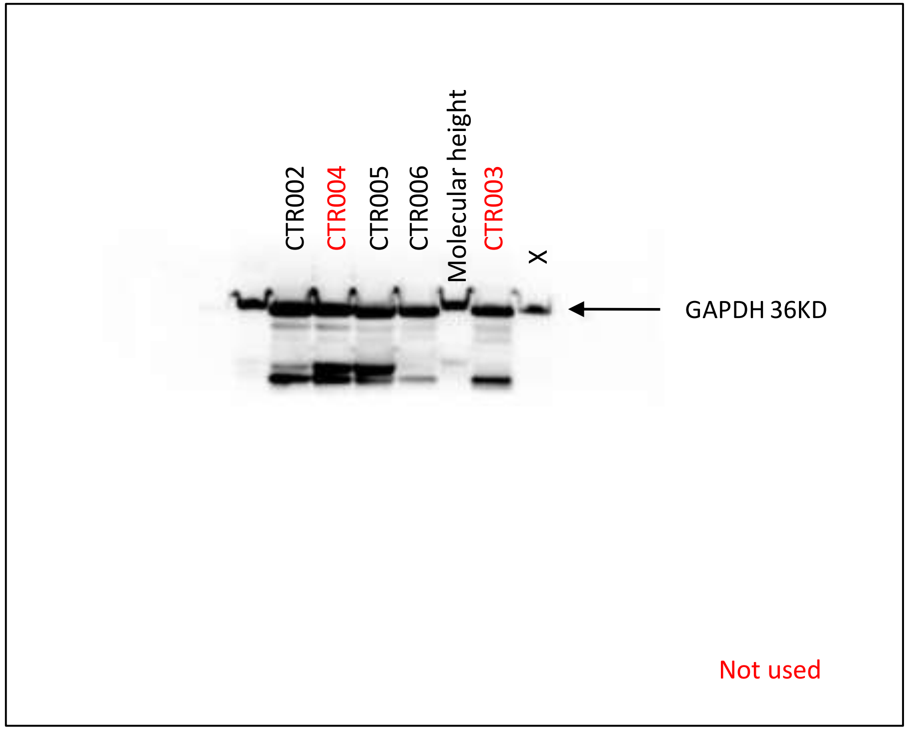
CTR002
CTR004
CTR005
CTR006
Molecular height
CTR003
X
GAPDH 36KD
Not used

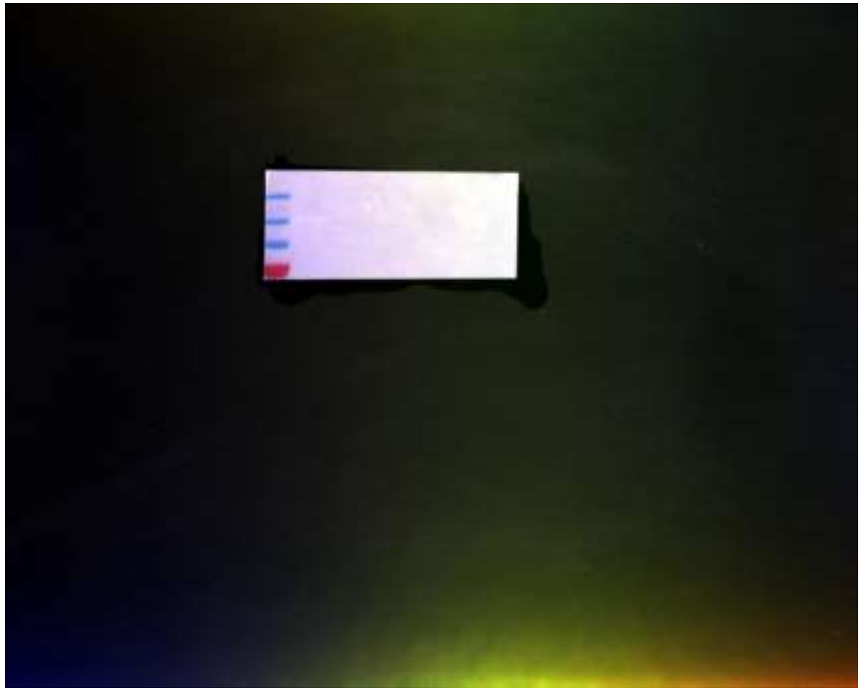

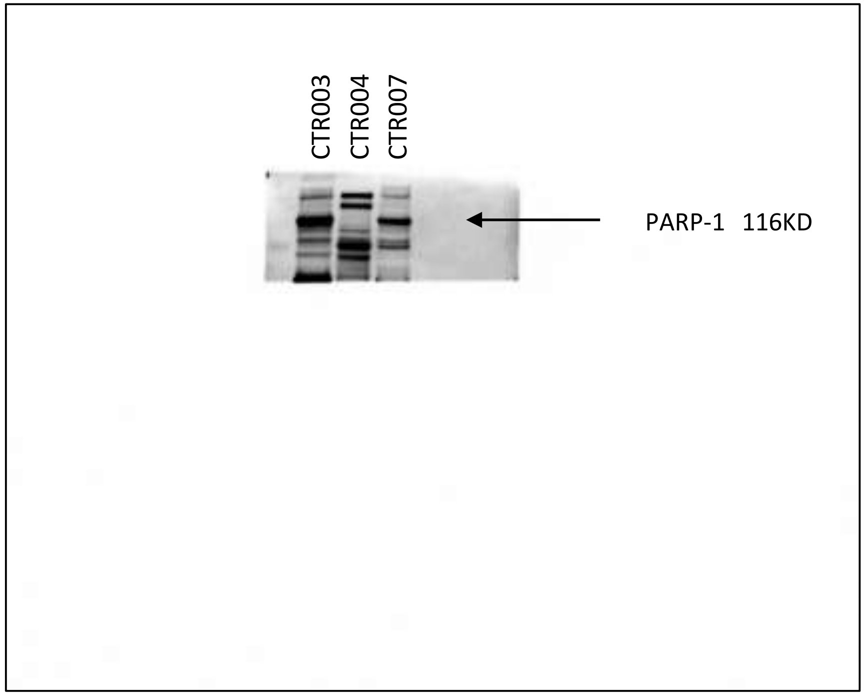

CTR003
CTR004
CTR007
PARP-1 116KD

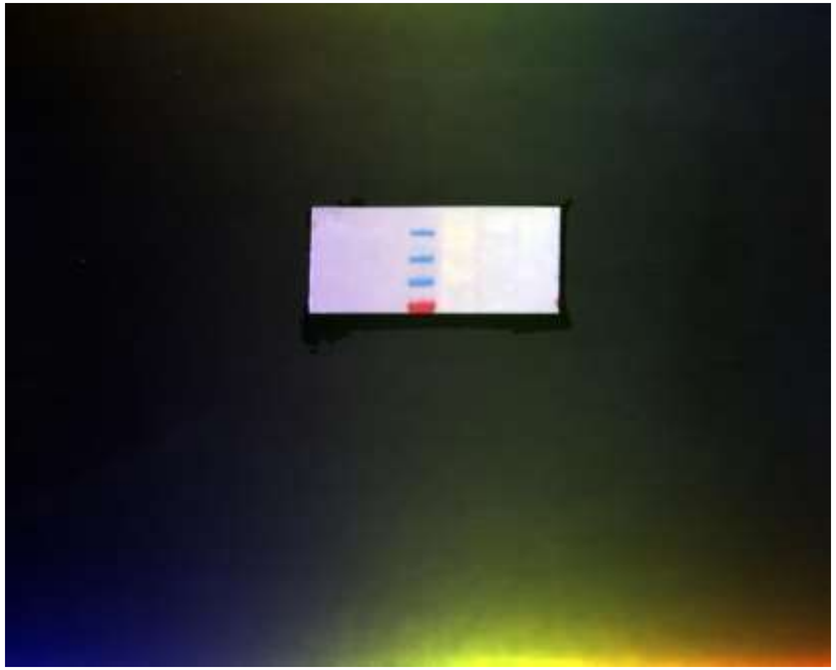

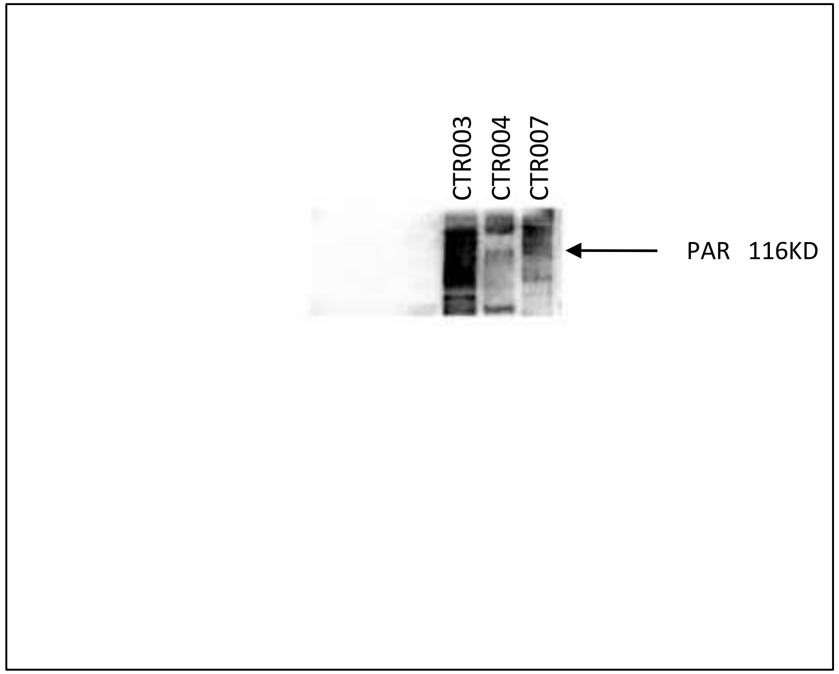

CTR003
CTR004
CTR007
PAR 116KD

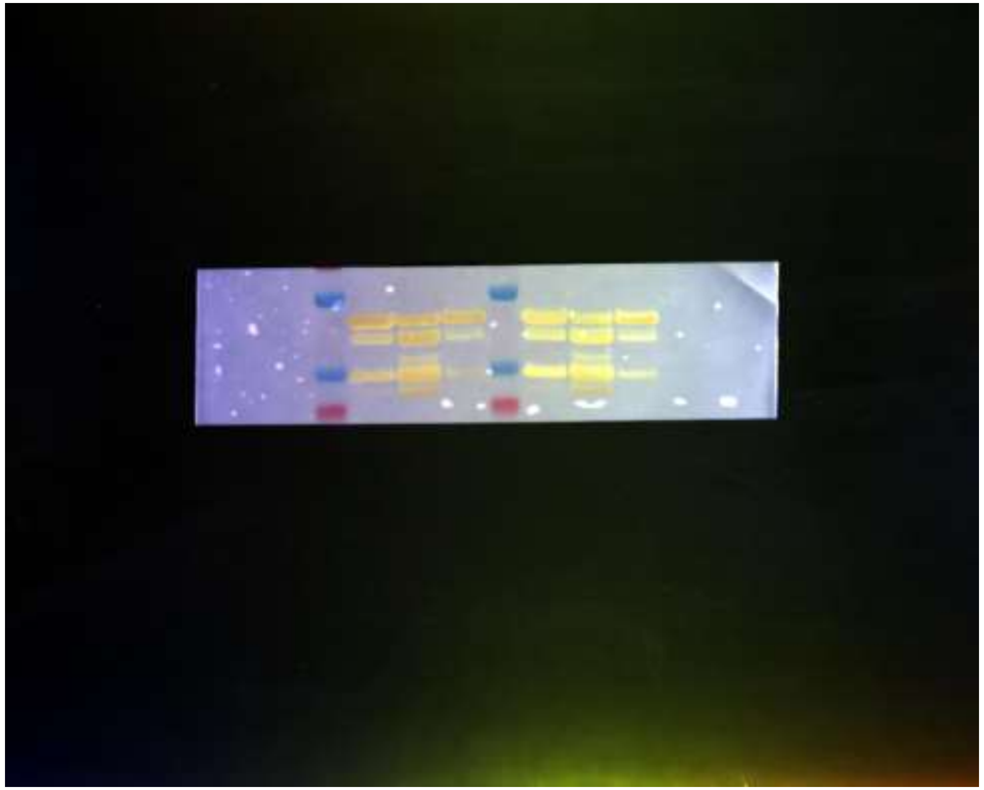

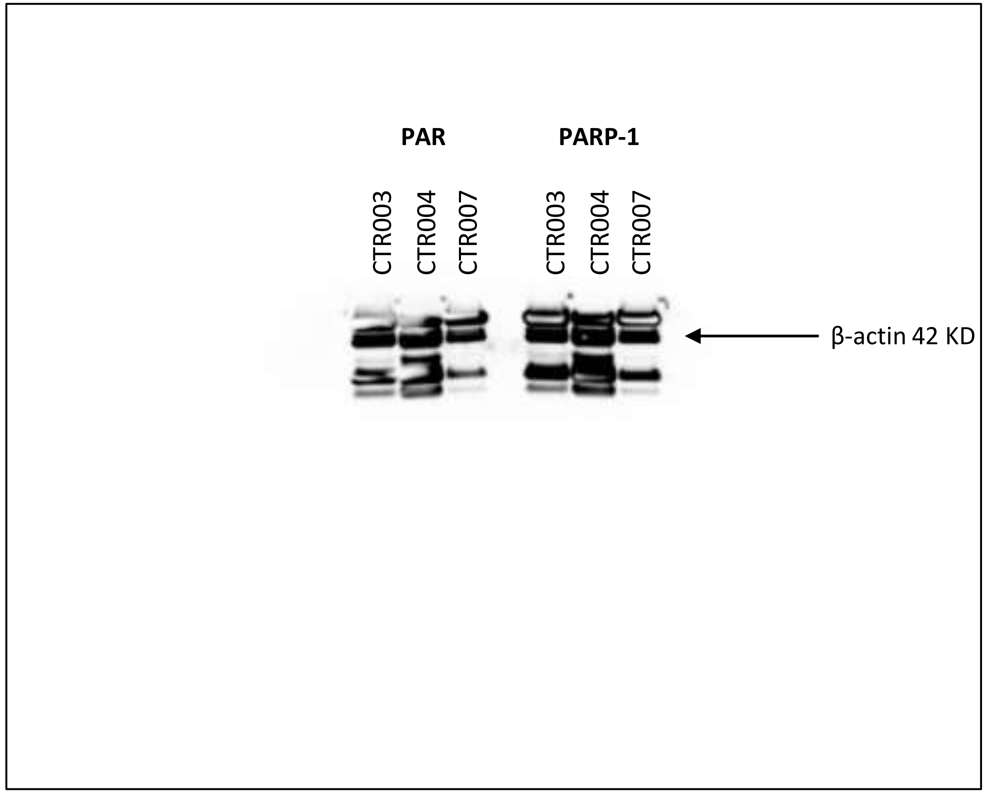

PAR
PARP-1
CTR003
CTR004
CTR007
CTR003
CTR004
CTR007
β-actin 42 KD

Not used

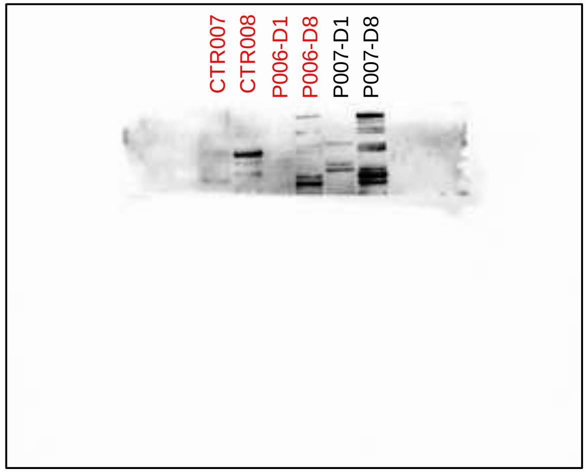
CTR007
CTR008
P006-D1
P006-D8
P007-D1
P007-D8

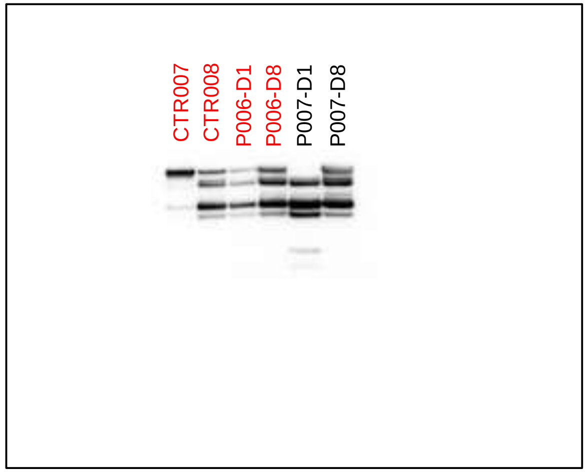
CTR007
CTR008
P006-D1
P006-D8
P007-D1
P007-D8

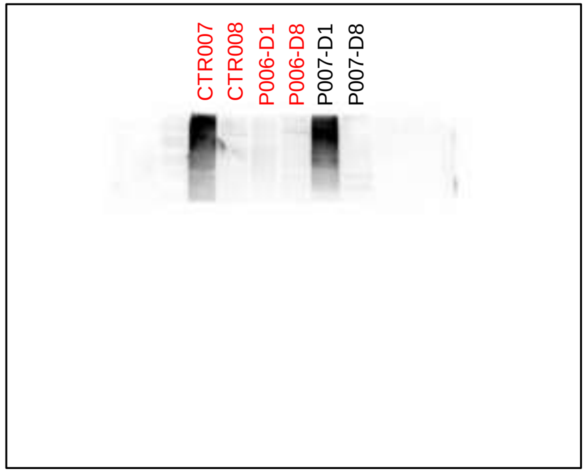
CTR007
CTR008
P006-D1
P006-D8
P007-D1
P007-D8

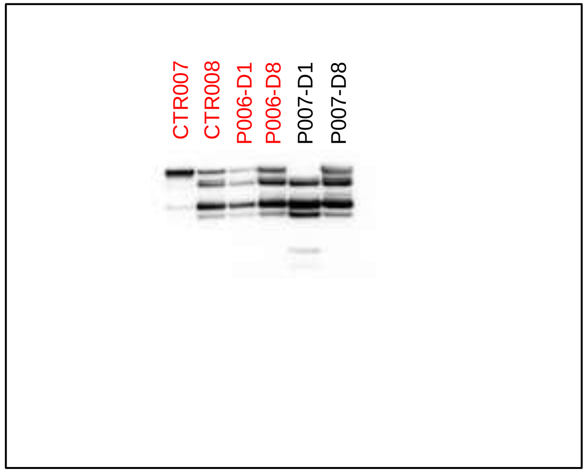
CTR007
CTR008
P006-D1
P006-D8
P007-D1
P007-D8

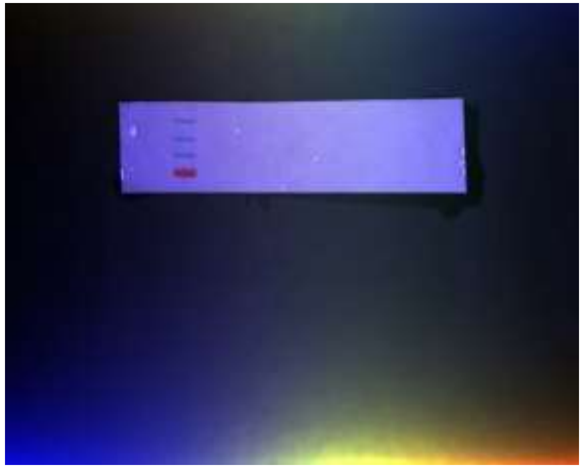

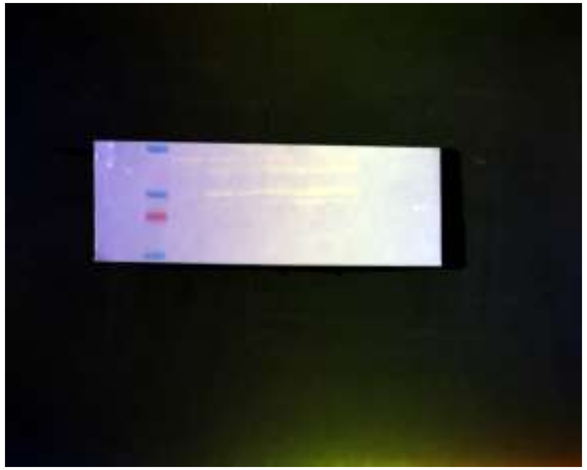

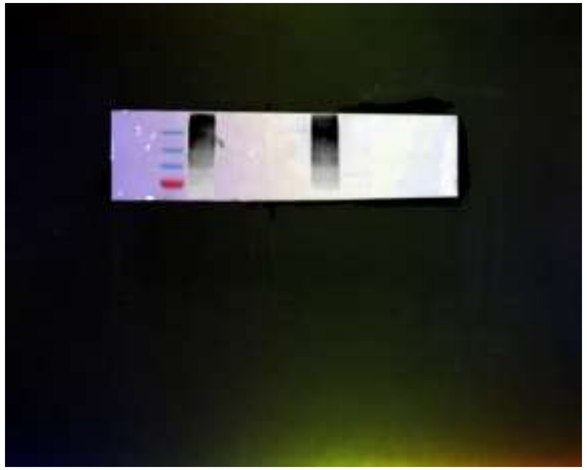

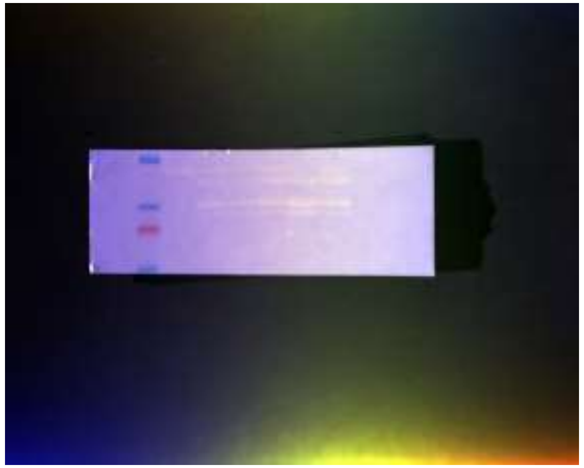

Paper Figure

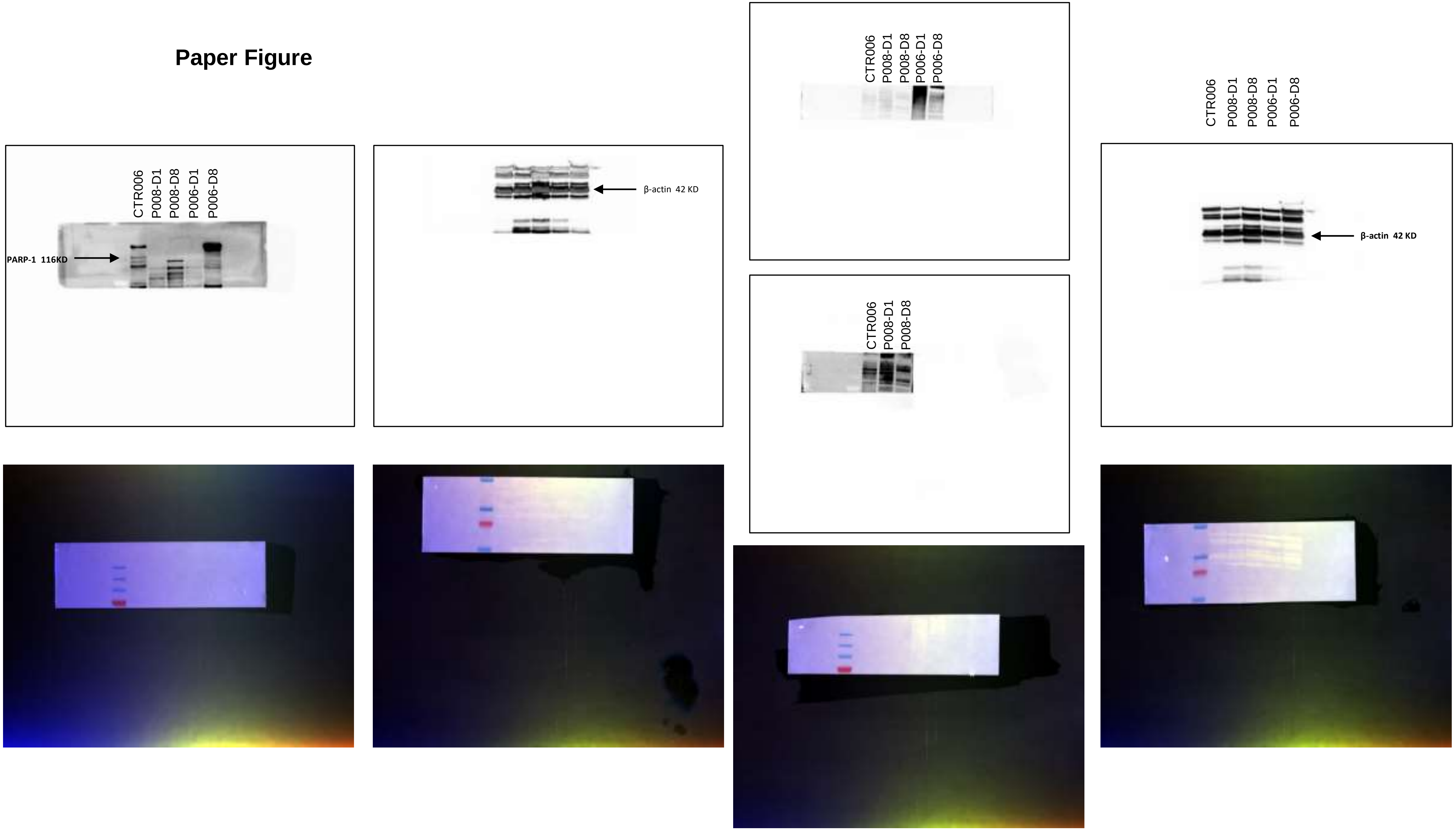

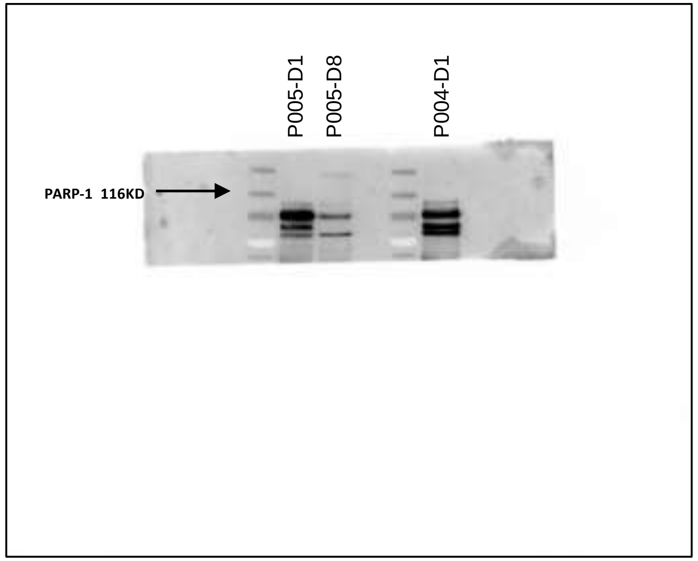
P005-D1
P005-D8
P004-D1
PARP-1 116KD

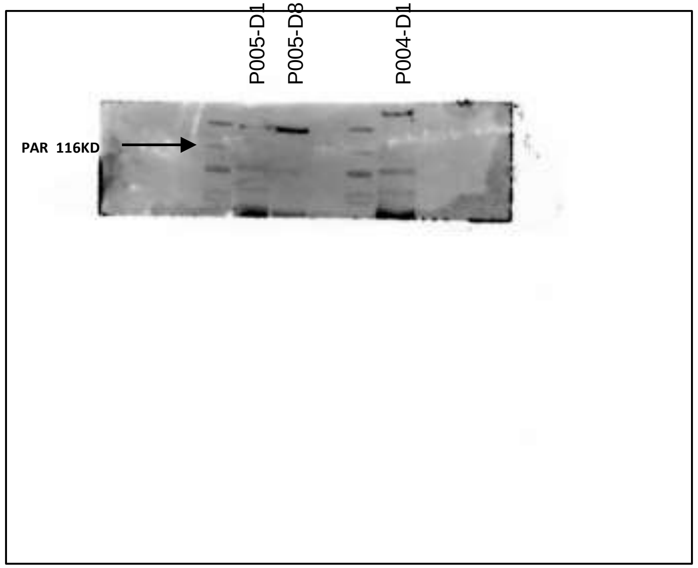
P005-D1
P005-D8
P004-D1
PAR 116KD

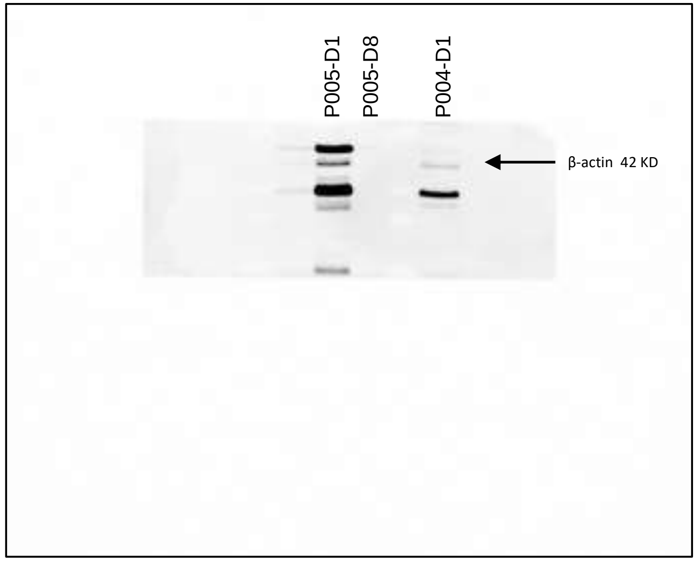
P005-D1
P005-D8
P004-D1
β-actin 42 KD

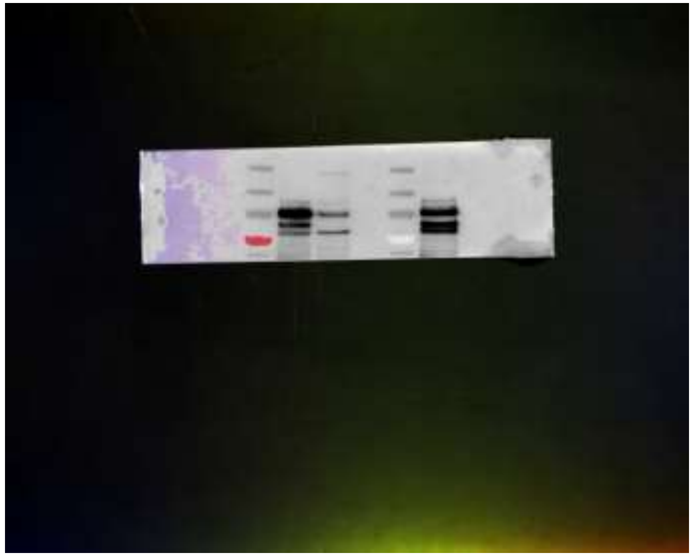

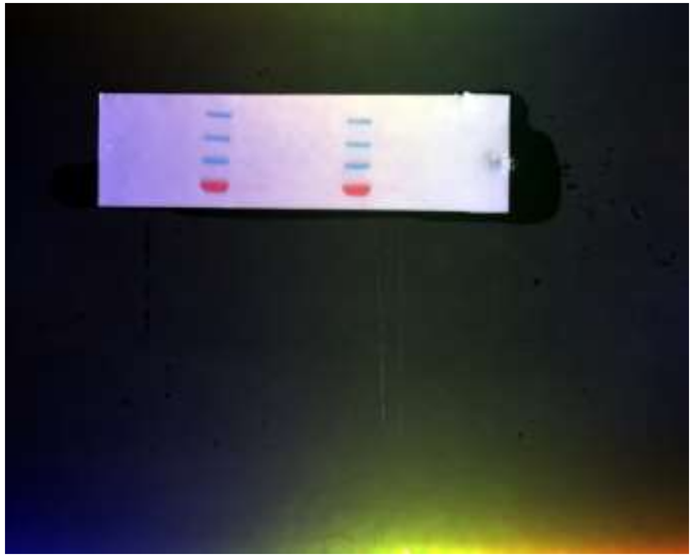

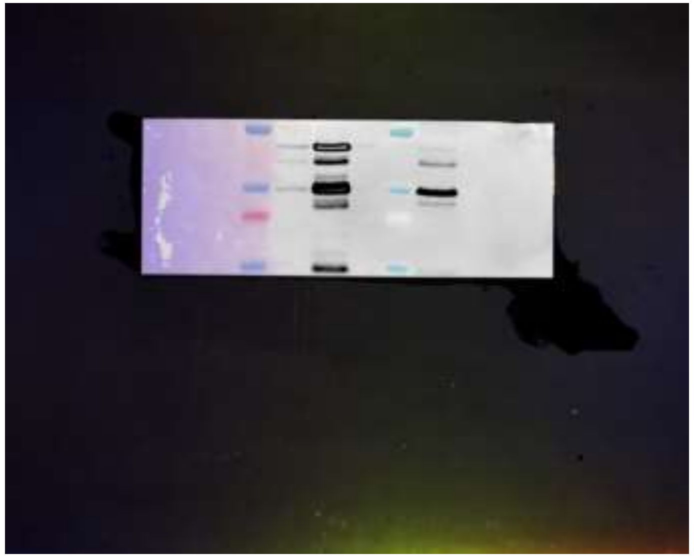

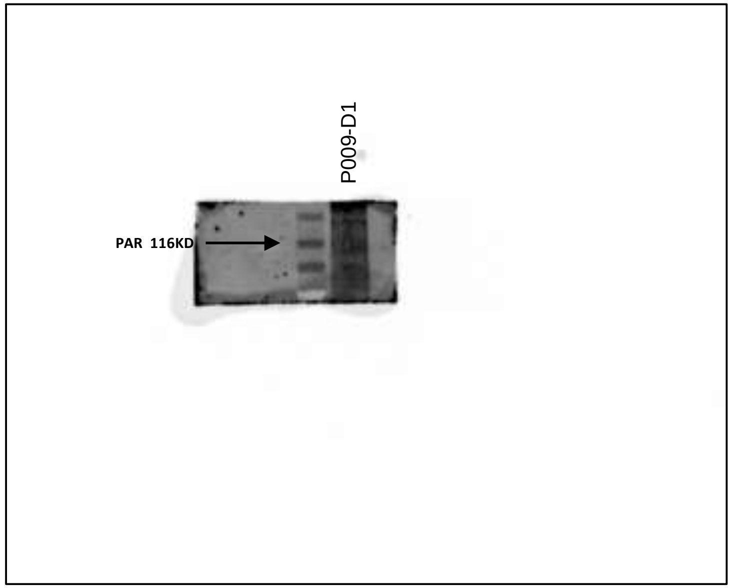
P009-D1
PAR 116KD

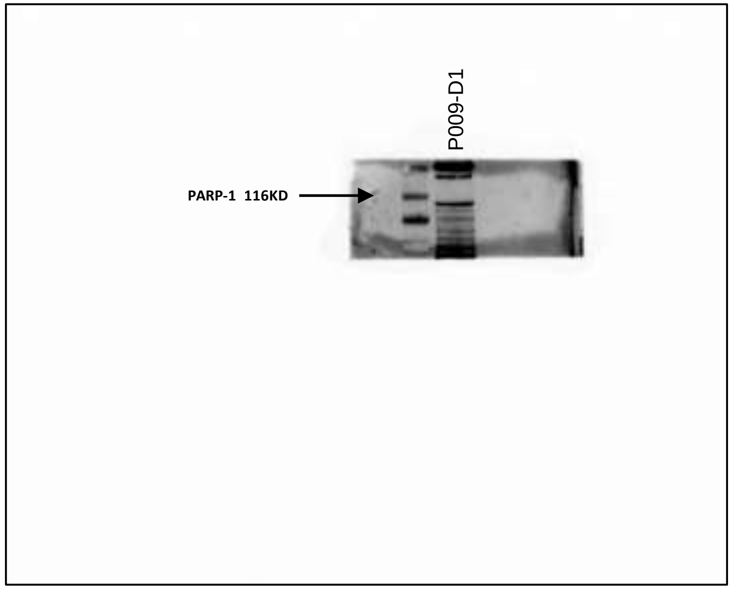
P009-D1
PARP-1 116KD

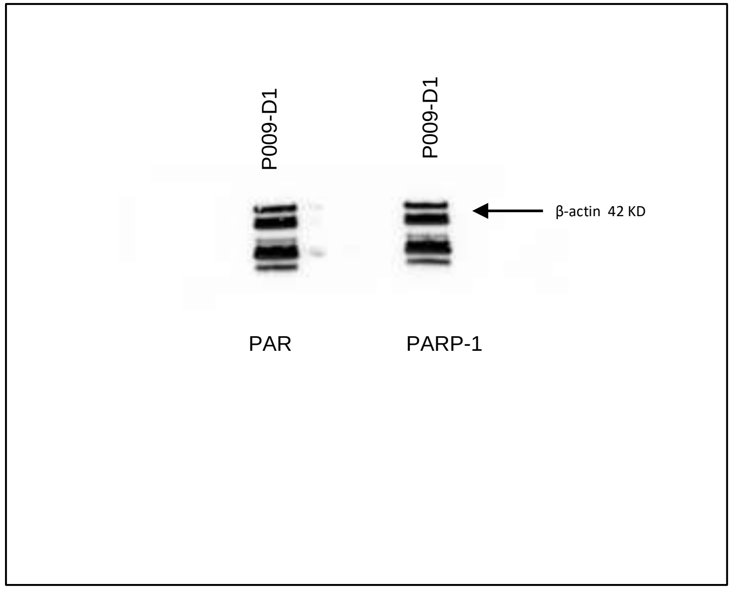
P009-D1
P009-D1
β-actin 42 KD
PAR
PARP-1

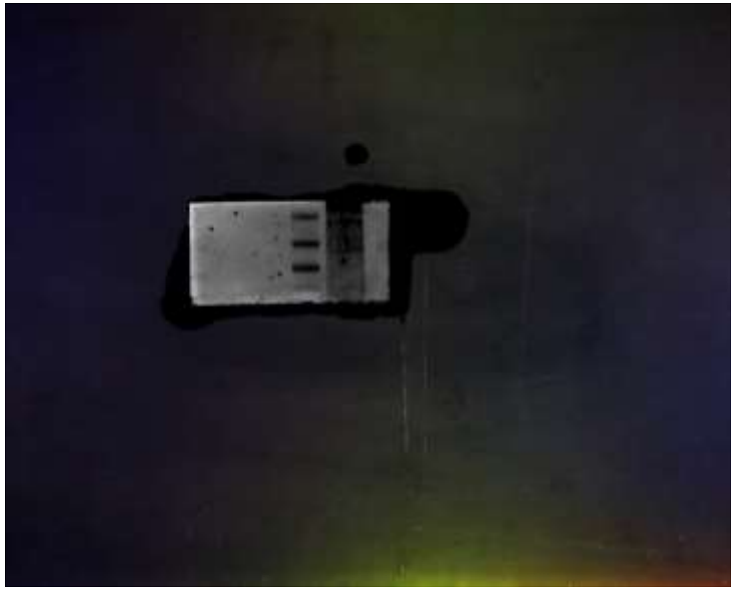

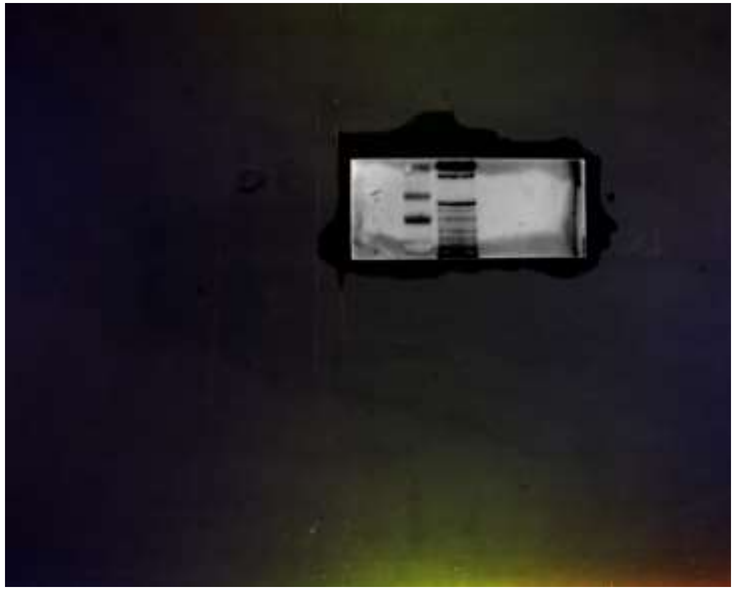

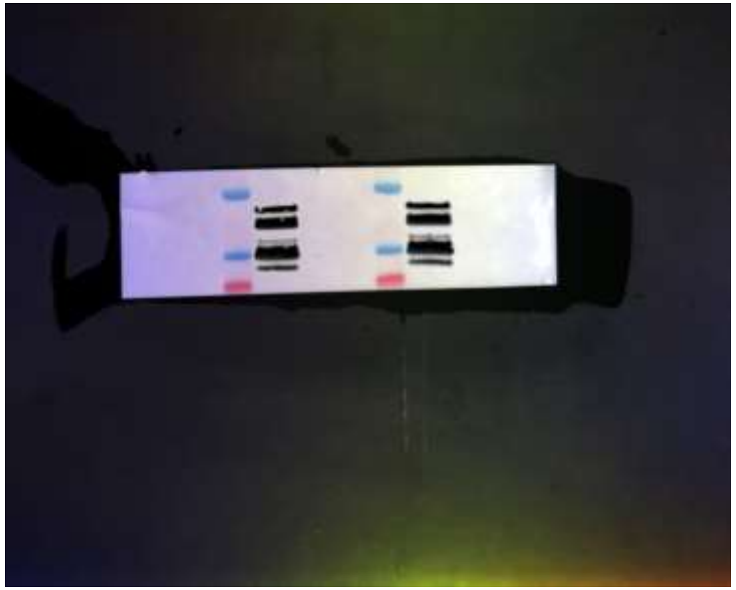

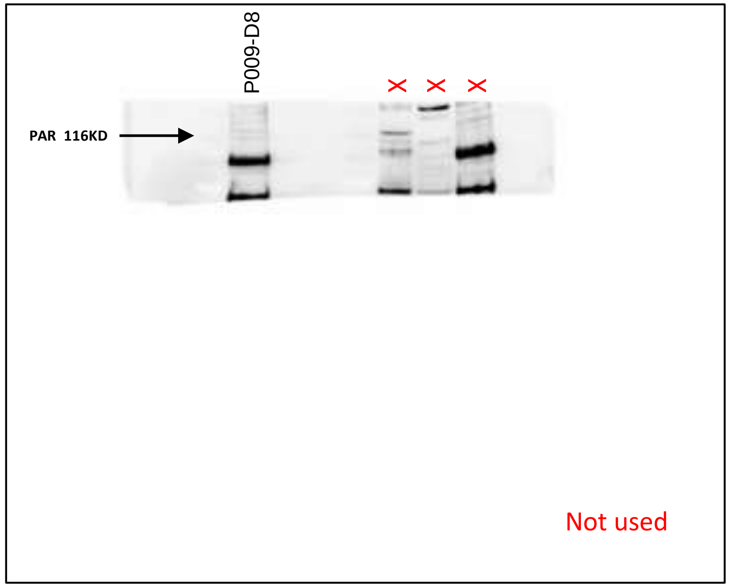
P009-D8
PAR 116KD
Not used

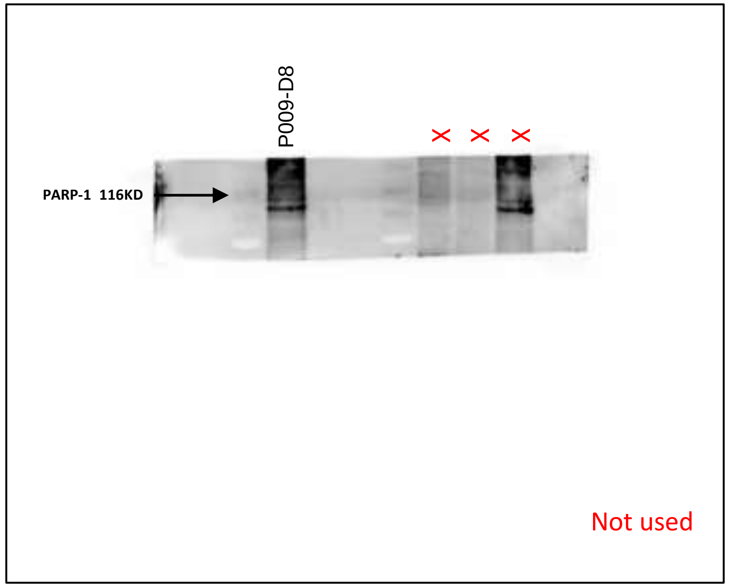
P009-D8
PARP-1 116KD
Not used

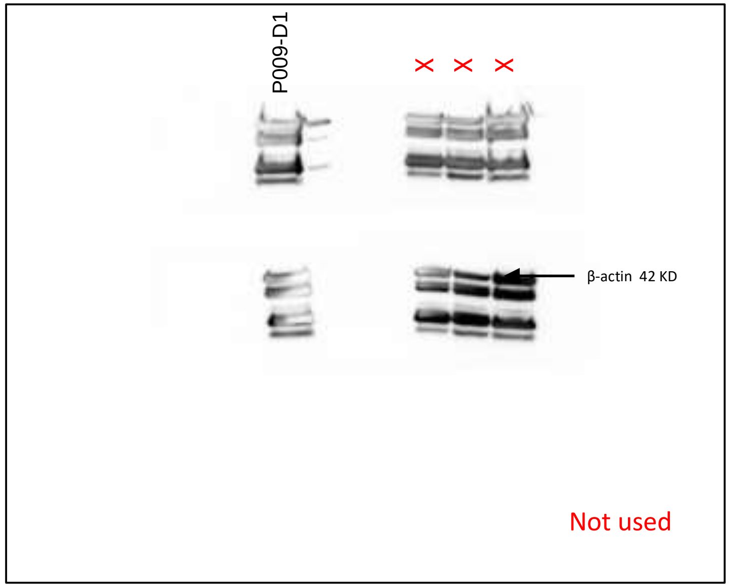
P009-D1
β-actin 42 KD
Not used

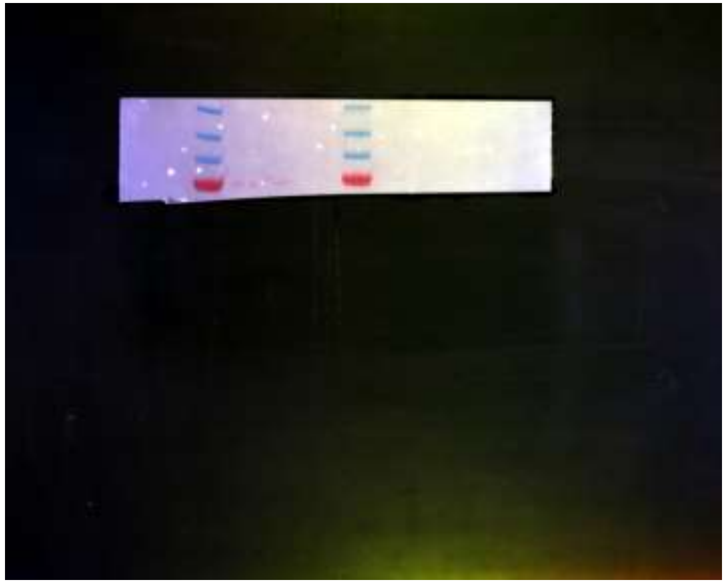

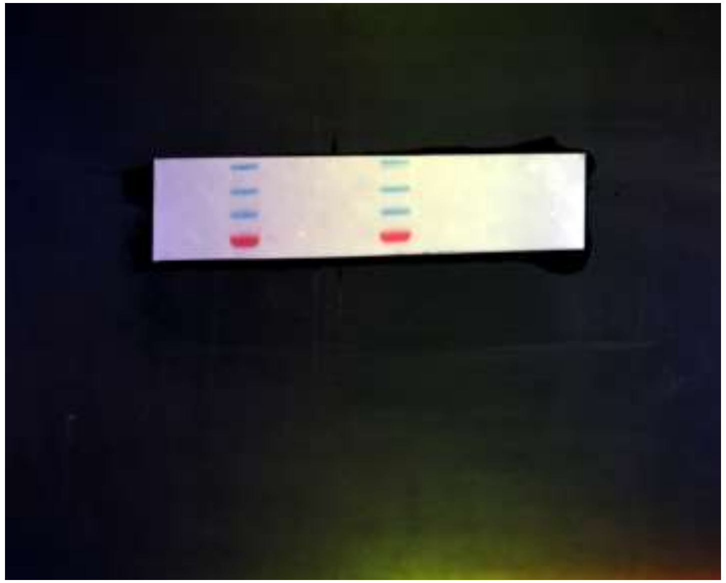

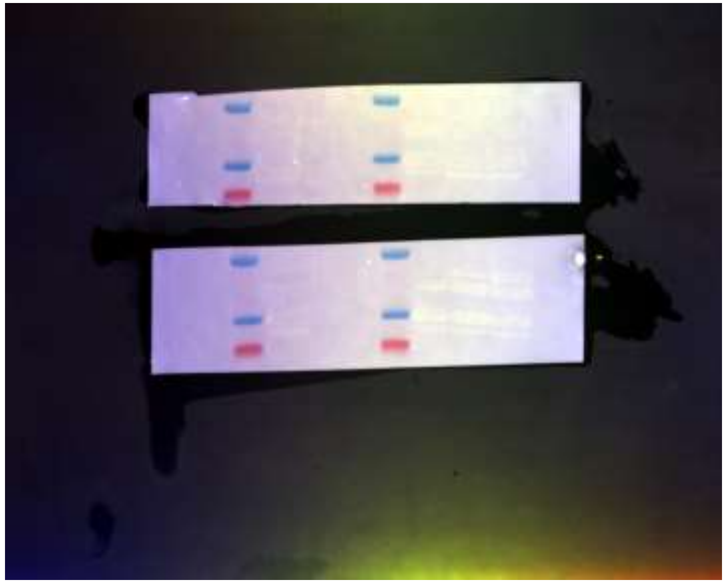

Supplement: Supplementary file 1 — Supplementary Material 1. [file 12931_2026_3623_MOESM1_ESM.pdf]
